# Genetic control of stem elongation in common bean and the influence of age and flowering time

**DOI:** 10.1007/s00122-025-04996-8

**Published:** 2025-08-21

**Authors:** Chantelle J. Beagley, Jacqueline K. Vander Schoor, Jakob B. Butler, James L. Weller

**Affiliations:** 1 School of Natural Sciences, University of Tasmania, Private Bag 55, Hobart, TAS 7001 Australia; 2 ARC Centre of Excellence for Plant Success in Nature and Agriculture, University of Tasmania, Hobart, TAS 7001 Australia

## Abstract

**Key message:**

Genetic analysis of stem elongation in common bean identified loci acting at different developmental stages and reveals interactions with flowering time.

**Abstract:**

Stem internode elongation is a major determinant of growth habit and plant height, and is often responsive to environmental signals, making it an integral component of plant architecture and a core characteristic targeted for crop improvement. More effective exploitation of stem elongation requires increased understanding of not only major determinants, but also of more subtle, specific influences as breeders seek to further refine adaptation to local growing environments. Common bean is a globally important legume crop and exhibits extensive variation in stem elongation, but there is little knowledge about the underlying genetic control. To better understand this trait, we performed a comprehensive genetic analysis in a wide F_2_ cross between wild and domesticated bean. By conducting quantitative trait locus (QTL) analyses of the length of individual internodes, we discovered four main genomic regions influencing internode length at different stages of development. Of these, one demonstrated strong association with the two known flowering genes *Fin* and *Ppd* on chromosome 1, while two other regions were validated in subsequent F_3_ and F_4_ generations and were demonstrated to be independent of flowering time. These results highlight the complex dynamic nature and potential pleiotropic interactions of elongation genes throughout development, and indicate new avenues of inquiry towards improving crop adaptation to specific environments.

**Supplementary Information:**

The online version contains supplementary material available at 10.1007/s00122-025-04996-8.

## Introduction

Plant architecture is a term used to refer to the overall appearance of a plant, and is determined by the number, size, shape, orientation, and distribution of individual plant organs. For the plant shoot, this includes “vegetative” traits such as branching and stem (internode) elongation, as well as reproductive-stage traits such as onset and duration of flowering, and inflorescence structure. These traits can contribute to plant architecture separately but may also interact, as seen most profoundly in rosette species which undergo rapid stem elongation or “bolting” at the same time as flowering. Environmental factors such as daylength, light quality and temperature also impact plant architecture (Chen 2019; Ohtaka et al. 2020; van den Ende and Zeevaart 1971; Wu et al. 2025), and the plasticity of these component traits allows plants to respond to their environment, reflecting the importance of plant architecture to adaptation.

Genetic variation in plant architecture has long been recognized and utilized in crop breeding. One simple and well-known illustration is the use of single gene “dwarfing” mutations in cereals during the green revolution of the 1960s, which significantly improved lodging tolerance and resource partitioning (Hedden 2003; Peng et al. 1999). While this presents a rather basic example in which plant architecture improved crop performance through a single gene, genetic variation for different component traits may also interact to affect many other aspects of crop performance in a more complex manner. For example, the interaction of internode elongation and the number, angle and length of branches helps determine canopy structure, which ultimately influences light interception, disease susceptibility and evapotranspiration, and influences harvestability through effects on pod height (Farooq et al. 2019; Kuzbakova et al. 2022; McDonald et al. 2013; Murchie and Burgess 2022). To better predict crop performance, a greater understanding of these intricate trait interactions is needed, and one way this can be established is through insights into the genetic control of individual component traits.

Stem elongation is arguably one of the most important of these component traits, as it both determines and interacts with various aspects of plant architecture throughout development, subsequently influencing seedling establishment, competitive ability and yield distribution (Beagley and Weller 2024). A refined understanding of internode length control is increasingly being considered important to improve lodging tolerance, enable dense planting, and ultimately increase yield in legumes (Beagley and Weller 2024; Fang et al. 2024). However, to effectively implement this strategy, knowledge of genes which specifically target elongation is needed.

The fundamental genetic basis of stem elongation is relatively well understood, primarily from study in model systems such as Arabidopsis, cereals, and pea (Beagley and Weller 2024; Wang et al. 2018), in which major genes, most prominently gibberellin biosynthesis genes, have been identified (Lester et al. 1997; Rieu et al. 2008; Spielmeyer et al. 2002). Many of these have a pervasive effect on stem elongation throughout development, but others may have a more prominent influence at specific developmental stages (Beagley and Weller 2024). Such loci are less likely to be detected from a single general measurement of stem length but may allow for more targeted adjustment of elongation.

Common bean (*Phaseolus vulgaris)* is one of the most important crop legumes for direct human consumption and is consumed worldwide (Hayat et al. 2014; Lisciani et al. 2024). Whereas wild forms exhibit an obligate short-day requirement for flowering, profuse branching and an indeterminate climbing habit with numerous elongated internodes (Kelly 2001; Toro et al. 1990), cultivated forms show a wider range of growth habits, more variable internode elongation, and reduced photoperiod sensitivity (Kelly 2001; Kwak et al. 2012). Changes in plant architecture have been crucial in the domestication and diversification of common bean, such as the adoption of determinacy at higher latitudes to adapt to the shorter growing conditions (Gepts 2014; Gepts and Debouck 1991; Kwak et al. 2012; Smartt 1990). However, the direct contribution of stem elongation to bean domestication is unknown, although modifications to elongation might plausibly be beneficial in different circumstances; for example, through increased elongation to improve first pod height, or reduced elongation to control lodging. While the genetic control of elongation has been considered in several studies and a handful of QTL identified (Checa and Blair 2008; González et al. 2016; Koinange et al. 1996), there have been no detailed studies of internode elongation, and in particular, in relation to how it may vary through development.

In contrast, the genetic control of flowering and determinacy in common bean has been examined far more extensively, leading to the identification of several major genes. Determinacy is primarily governed by the *Fin* gene *(TERMINAL FLOWER 1, TFL1*) (Kwak et al. 2012; Kwak et al. 2008; Repinski et al. 2012), while photoperiod sensitivity is conferred by *COL2* (González et al. 2021) and *Ppd (PHYTOCHROME A3*, *PHYA3*) (Gu et al. 1998; Wallace et al. 1993; Weller et al. 2019). Although these genes play a significant role in common bean evolution, investigation into their interactions with other developmental pathways is needed for a more complete understanding of plant development and adaptation.

This study aims to explore the genetic control of elongation in common bean, using an F_2_ population of a wide cross previously shown to vary for major genes in several components of plant architecture including determinacy, twining habit, total node number (e.g. 5–15), length of fifth internode (e.g. 27–40 mm) and flowering time (DTF 32—over 130 days) (González et al. 2021; Koinange et al. 1996; Repinski et al. 2012; Weller et al. 2019). This provides the additional opportunity to examine the interaction between these traits. Most previous studies of elongation in common bean only considered a single representative internode, but in this study we have taken the more detailed approach of conducting QTL analysis of each successive internode. This has allowed a comprehensive assessment of genetic variation for elongation and its interactions across the lifespan of the plant.

## Materials and methods

### Experimental material and measurements

Flowering time variation has been investigated previously in the F_2_ of a biparental cross between the indeterminate Mesoamerican wild accession G12873, and the determinate domesticated Andean accession, cv. Midas (Weller et al. 2019). To examine the genetic control of elongation and the potential interaction with flowering, an additional population of 300 F_2_ individuals from the same cross was grown in a temperature-limited glasshouse at the University of Tasmania in Hobart and maintained under non-inductive long-day photoperiod conditions (18 hour light, 6 hours dark) consisting of natural light extended with sodium vapour lamps before dawn and after dusk at an irradiance of ~50 μmol m^-2^ s^−1^. Under these same conditions, subsequent F_3_ and F_4_ populations segregating for identified Chr07 and Chr04 QTL regions were also grown for QTL validation (See QTL validation section).

Internode length was measured as the distance between nodes on the main stem and recorded with a ruler for every internode at the end of the life cycle for all F_2_ individuals. The distance from the cotyledon to first node was measured and denoted as internode 1 (I1). Measurements continued until the plants terminated. The node of floral initiation was also recorded (NFI).

### DNA extraction and DArTseq genotyping

Young leaf tissue from the parents and each F_2_ individual were harvested and ground using mortar and pestle under liquid nitrogen. Total genomic DNA was extracted using the standard CTAB method (Doyle and Doyle 1987). DNA samples from 274 individuals were standardised to 50 ng/µl concentration using sterile Milli-Q water and sent to Diversity Arrays Technology Pty Ltd (Canberra, Australia) for genotyping. DArT markers were generated and genotyped in the F_2_ population using the high-throughput DArTSeq™ method (Jaccoud et al. 2001; Sansaloni et al. 2011), which creates a 64 bp sequence marker through Next Generation Sequencing (NGS).

Two types of markers were received from DArT: co-dominant SNP markers (two doses or one dose or absent), and dominant, biallelic markers (presence/absence SilicoDArTs). Markers were ranked on the basis of quality (from 1-high to 4-low) according to parameters provided with the DArT dataset and the criteria outlined in Supplementary Table S1, dependent on whether they were a dominant or co-dominant marker. Markers that did not meet these standards were excluded from further analysis.

### Linkage map construction

The vast number of markers generated from high-throughput genotyping-by-sequencing technologies such as DArT imparts a high computational load and challenges conventional mapping processes (Jighly et al. 2015; Ronin et al. 2010). Given computational limitations and to improve the accuracy of the constructed map, an initial skeleton or ‘bin’ map was developed from a subset of markers decided through a ‘binning’ process in SimpleMap (Collard et al. 2009; Jighly et al. 2015), this reduced marker number from 4330 to 487. This process involves grouping closely linked markers on the basis of a user-defined maximum recombination threshold (repulsion threshold) and reduces the number of markers required to be ordered in the linkage map analysis software at once*.* SimpleMap recommends a maximum repulsion threshold equivalent to 3 cM for maps constructed with the Kosambi mapping function (Jighly et al. 2015), which corresponds to a recombination frequency of ~3% for small map distances (Sturtevant 1913). Therefore, for the G12873 x Midas F_2_ population (*n*=300) a repulsion threshold of 9 (i.e. any markers with 9 or more recombinants between them were not binned together) was established.

During the binning process a single marker was selected to represent all the markers in that bin. These representative markers were then assigned into linkage groups using *JoinMap* v4.0 (Van Ooijen 2006) to construct the initial skeleton map. Markers were placed into groups that gathered at a minimum *Logarithm of Odds score* (LOD) of 3, as conventionally a LOD score of >3 is regarded as significant linkage (~*P*=0.05) (Nyholt 2000). Representative markers were then ordered within each linkage group using the regression mapping algorithm in *JoinMap*, employing the Kosambi mapping function (Kosambi 1943). To improve marker order robustness and map accuracy, representative markers were ordered through an iterative mapping procedure from highest to lowest quality (rank 1 to rank 4) following the technique employed by (Butler et al. 2017). In each iteration, problematic markers were identified as per the following criteria: markers with a chi-square goodness-of-fit > 3.0 (poor goodness-of-fit = large chi-square values), or present in any significant double cross-over events or were in ≥10 non-significant double cross over events. Markers which exhibited drastically different segregation distortion from their closely-linked markers were also flagged as problematic, as they were likely to represent genotyping errors (Van Ooijen 2006). Each conflicting marker was removed (excluding lower quality markers in the case of conflicts), and the linkage map was re-calculated. This procedure was repeated until all conflicting markers were excluded in each linkage group, resulting in the initial skeleton map which consisted of 311 representative markers.

To produce the ‘Comprehensive’ final map all markers from the binning procedure were reintegrated back into the initial skeleton map, resulting in a total of 3286 markers present in the comprehensive linkage map. This was performed using SimpleMap, with binned markers being inserted around the fixed representative marker using the percentage of recombinants between two markers (Jighly et al. 2015). The marker sequences (64 bp) from the markers used for the skeleton map were queried against the *Phaseolus vulgaris* v2.1 reference genome (Schmutz et al. 2014) using BLASTN (Altschul et al. 1997) to evaluate synteny. Markers above the threshold of acceptance (e-value ≤ 2.96E-06) were able to be positioned in the bean reference genome assembly. Generated linkage maps were visualized using MapChart v2.2 (Voorrips 2002).

### QTL analysis and discovery of positional candidate genes

QTL analysis was undertaken with *MapQTL* v6.0 (Van Ooijen 2009) using the regression approximation to maximum likelihood mapping (Haley and Knott 1992; Martinez and Curnow 1992) in conjunction with the less-dense skeleton map, as it provided adequate marker distribution and map coverage while substantially reducing the computational demand (Van Ooijen 2009). Additionally, to enhance our ability to create relevant boundaries for candidate gene evaluation, single markers found non-syntenic with the reference genome were removed from the map prior to QTL analysis.

Appropriate significance thresholds for QTL detection were determined by permutation tests at genome-wide and chromosome-wide levels (1000 permutations) (Churchill and Doerge 1994). Putative QTL were defined at two different levels; suggestive (chromosome-wide type I error rate <0.05); and significant (genome-wide type I error rate <0.05). QTL were identified through an iterative approach, in which an initial round of interval mapping function was applied to determine marker co-factors. For each putative QTL that exceeded the suggestive significance (chromosome-wide) threshold, the DArT-markers closest to the peak (highest LOD score) were selected as cofactors for subsequent restricted multiple QTL mapping (rMQM) and MQM mapping. The rMQM and MQM functions were reiteratively performed until no further QTL exceeding the significance thresholds were detected and QTL positions were stable. QTL were named according to the internode they correspond to and an indication of their rank in importance based on their respective LOD scores (e.g. I5.1 represents the internode 5 QTL with the largest LOD score). 2-LOD confidence intervals were established around each internode QTL, and the markers flanking these intervals were used to position these QTL in the *Phaseolus vulgaris* v2.1 reference genome (Schmutz et al. 2014).

Annotated genes within these intervals (or combined intervals in the case of collocated QTL clusters) were selected as putative positional candidate genes for the associated traits. Relevance of these genes were assessed through examination of literature on the genetic control of internode elongation.

### QTL validation: fine mapping and HRM analysis

Appropriate segregating F_3_ populations for the Chr04 and Chr07 QTL regions were developed through selection of individuals from the F_2_ population that were heterozygous at the strongest putative QTL region and fixed for the second strongest QTL and subsequent QTL where possible (Supplementary Table S2 and S3). Additionally, the F_2_ population was genotyped for the known flowering time genes *Fin, Ppd* and *COL2* (primers detailed in Supplementary Table S4), and these loci were subsequently fixed for the functional wild-type in an effort to disentangle the effects of flowering time on these QTL regions. Two subsequent F_4_ populations were developed for the Chr07 population through selecting specific F_3_ individuals fixed across different parts of the QTL region.

Internode length from these populations were assessed in the same way as the F_2_ population, with the first 7 internodes measured in the F_3_ and subsequent F_4_ Chr07 populations, while length of internodes 7-17 were measured in the F_3_ Chr04 population. Using the bean genome (Schmutz et al. 2014), High resolution melt (HRM) markers were developed within the 2-LOD boundaries. Overall, 22 functional HRM markers were developed (Supplementary Table S5) and used to increase the mapping resolution within the Chr04 and Chr07 QTL regions. These markers were analysed and scored in the newly developed F_3_ populations using a Rotorgene Q HRM machine (Qiagen). T-tests (*P*≤0.05) assuming unequal variance were conducted to test for significant co-segregation between internode length variation and marker genotypes.

## Results

### Phenotypic variance

**Fig. 1:**
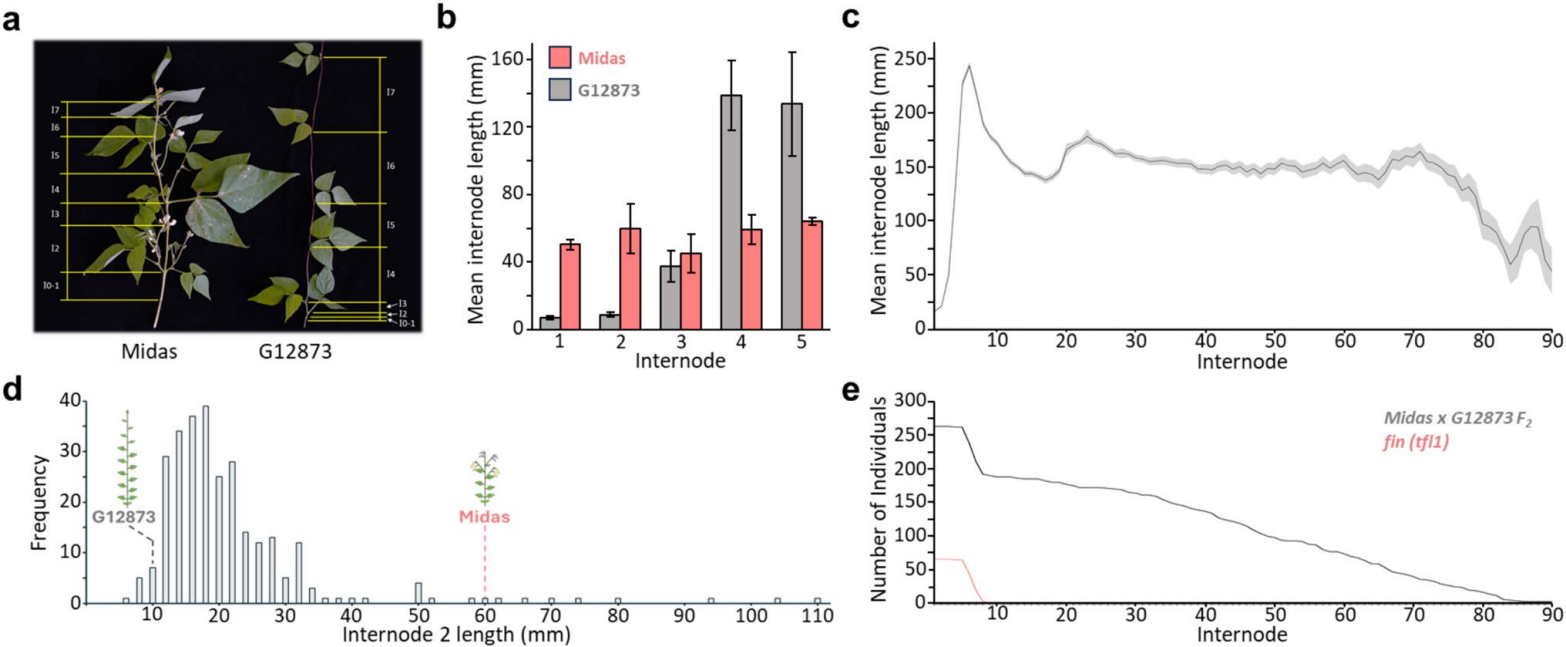
Differences in internode elongation observed in the parents and resulting F_2_ population of *Phaseolus vulgaris* grown under 18 h long-day (LD) conditions. **a** Visual differences in plant architecture and internode elongation in the domesticated (Midas) and wild (G12873) parents. **b** Differences in the mean internode length between the two parents. **c** Mean internode length across the F_2_ population (black line), the standard error for each internode is depicted in grey. **d** Distribution in the length of internode 2 across the subsequent F_2_ progeny. **e** The total number of individuals for which internode length could be measured at each internode (sample size). Displayed in red is the distribution of individuals in the F_2_ population with recessive *fin* loci, thus possessing a determinate growth habit

The wild (G12873) and domesticated (Midas) parents exhibited dramatic differences in several plant architecture traits, including determinacy, flowering, and elongation (Fig. 1a and b). Whereas G12873 possessed a thin stem and an indeterminate growth habit and did not flower under long-day conditions, Midas exhibited a thick stem, early flowering and a determinate main shoot. Previous investigations into flowering time variation have demonstrated that these parents differ at the shoot determinacy locus *Fin*/*TFL1y* (Repinski et al. 2012), and two photoperiod response loci, *Ppd/PHYTOCHROME A3* (*PHYA3*) and *CONSTANS-like 2 (COL2)* (González et al. 2021; Wallace et al. 1993; Weller et al. 2019), with Midas carrying recessive alleles at all three. The parents also exhibit clear differences in stem elongation (Fig. 1b). In G12873, the first few internodes were relatively short, with a steady increase in elongation over successive internodes. In contrast, the initial internodes in Midas were much longer but no further increase was observed.

Unsurprisingly, the F_2_ progeny grown under the same long-day conditions segregated for a number of these plant architecture differences, giving rise to a wide range of phenotypes and extensive variability. Variation for flowering time has previously been described in detail (Weller et al. 2019) but was also evident for stem elongation. For example, the length of internode 2 ranged from 6–110 mm, with a positively skewed distribution (Fig. 1d). Similarly, large variation in the lengths of individual internodes was seen throughout development (Fig. 1c; Supplementary Table S6). Across the progeny the mean lengths of the first 3 internodes were notably shorter, but elongation then increased dramatically over subsequent internodes to a peak at internode 6, before declining to a broadly consistent length of around 150 mm, with a further small peak around internode 23 (Fig. 1c; Supplementary Table S6).

Total internode number also varied across the F_2_ population, reflecting the extensive variation in flowering time, determinacy, and length of the reproductive phase across the population. This is evident in the decreasing number of individuals for which internode length could be measured (Fig. 1e). In effect this meant that the average internode length across development (as shown in Fig. 1c) was determined from a decreasing number of individuals as a growing proportion of the population reached maturity. In particular, the decrease in sample size preceding internode 10 was largely attributable to the fact that approximately one-quarter of the individuals were determinate (homozygous for the recessive *fin* allele) and had fewer than ten nodes at maturity.

### Linkage mapping and synteny with Phaseolus genome assembly

A linkage map for this F_2_ population was then constructed using 3286 markers, which spanned 1002.59cM in total (Supplementary Figure S1; Supplementary Table S7). Marker density was high, with a mean marker interval of 3.31cM and the largest gap spanning no more than 13cM. Of the 3286 markers ordered in the Phaseolus comprehensive map, 2723 were able to be positioned in the bean reference genome assembly, while 42 mapped to the minor scaffolds. The sizeable number of unplaced markers is unsurprising given our cross involves a wild parent, while the reference genome is of a domesticated accession. Synteny and co-linearity were very high, with only 4.96% of markers mapping to a different chromosome and no major rearrangements relative to the genome assembly (Supplementary Figure S1; Supplementary Table S7).

### QTL analysis

We next examined the genetic architecture of internode elongation in this population by performing a separate QTL analysis for each internode. QTL were identified for 56 of the 80 internodes analysed (70%), and from these an average of 2.7 QTL were identified per internode, ranging from 1 to 9 QTL (Supplementary Table S8; Supplementary Figure S2). Of these QTL, 68 were significant, and an additional 81 were suggestive, which were retained as many aligned together or with significant QTL. The identified QTL explained 2.7–51.6% of the phenotypic variance observed (PVE), with 52 QTL explaining ≥10% of the variance for their associated internode (Supplementary Table S8). Both wild and domesticated alleles conferred increased internode elongation at different QTL (Supplementary Table S8), but overall this was more common for the wild (G12873 and Heterozygote) allele (77% vs. 23%) (Supplementary Figure S3).

**Fig. 2:**
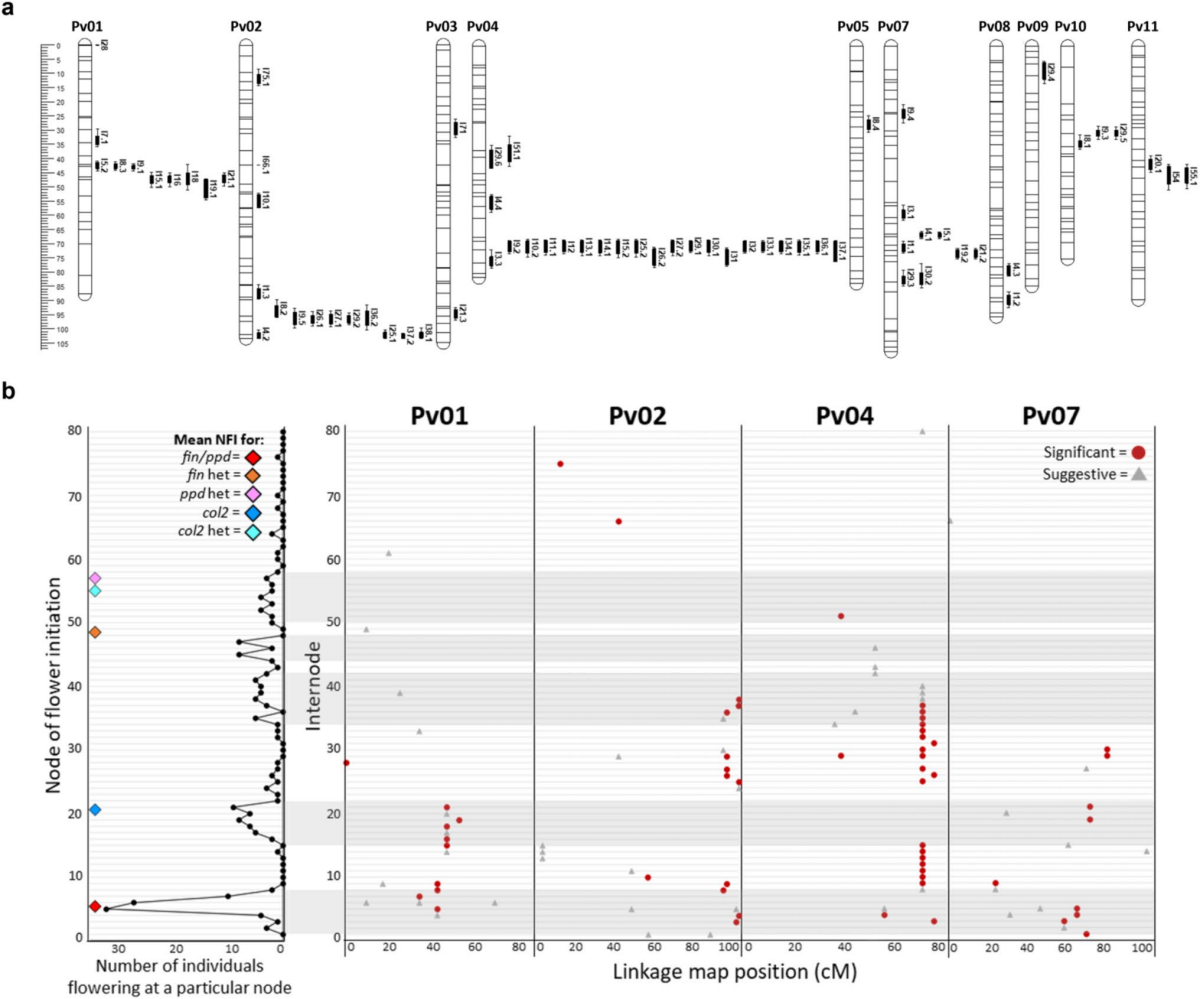
Distribution of detected internode length QTL in an F_2_ population derived from a cross between a wild (G12873) and domesticated (Midas) common bean.** a** Location of significant (genome-wide level) internode length QTL on the *Phaseolus vulgaris* skeleton linkage map. QTL are displayed to the right of each linkage group, with their respective internode number and relative LOD score ranking. One-LOD and two-LOD confidence intervals are denoted by the bars and *dashes* respectively. Horizontal lines on the linkage groups display marker positions. Figure created in MapChart v2.2 (Voorrips 2002). **b** The clustered regions on chromosome 1, 2, 4 and 7. On the left panel, the node of flower initiation (NFI) is plotted with the mean NFI for *fin, ppd* and *col2* individuals specified by the coloured diamonds. No *Fin/ppd* or *fin/Ppd* individuals were present in the population, so only a *fin/ppd* mean NFI is presented. In the right panel, significant QTL are specified by red circles, while suggestive QTL for internode length are denoted by the grey triangles

QTL distribution was not uniform across the 11 chromosomes, with tight co-location of numerous QTL observed on Chr01, Chr04, and to a lesser extent Chr02 and Chr07 (Fig. 2a; Supplementary Figure S2). These chromosomal regions each featured QTL that affected a different series of consecutive internodes, with QTL on Chr07 for internodes 1-5, on Chr02 for internodes 3-9 and 24-38, on Chr01 for internodes 4-9 and 14-21, and on Chr04 for internodes 8-15 and 25-40 (Fig. 2b). The alignment of these QTL is supportive of each region representing a single underlying locus except for the Chr01 region, in which the consistent shift in position for later internode QTL suggested two distinct loci (Fig. 2b). This is further supported by the largely consistent direction of the allelic effects of QTL within each chromosomal region (Supplementary Table S9). Interestingly, only the early internode region on Chr07 had increased elongation consistently conferred by the domesticated allele, with the exception of I4.6 and I5.2 on Chr01 (Supplementary Table S9), suggestive of selection for increased elongation specifically in early internodes.

### Identification of relevant candidate genes

**Fig. 3:**
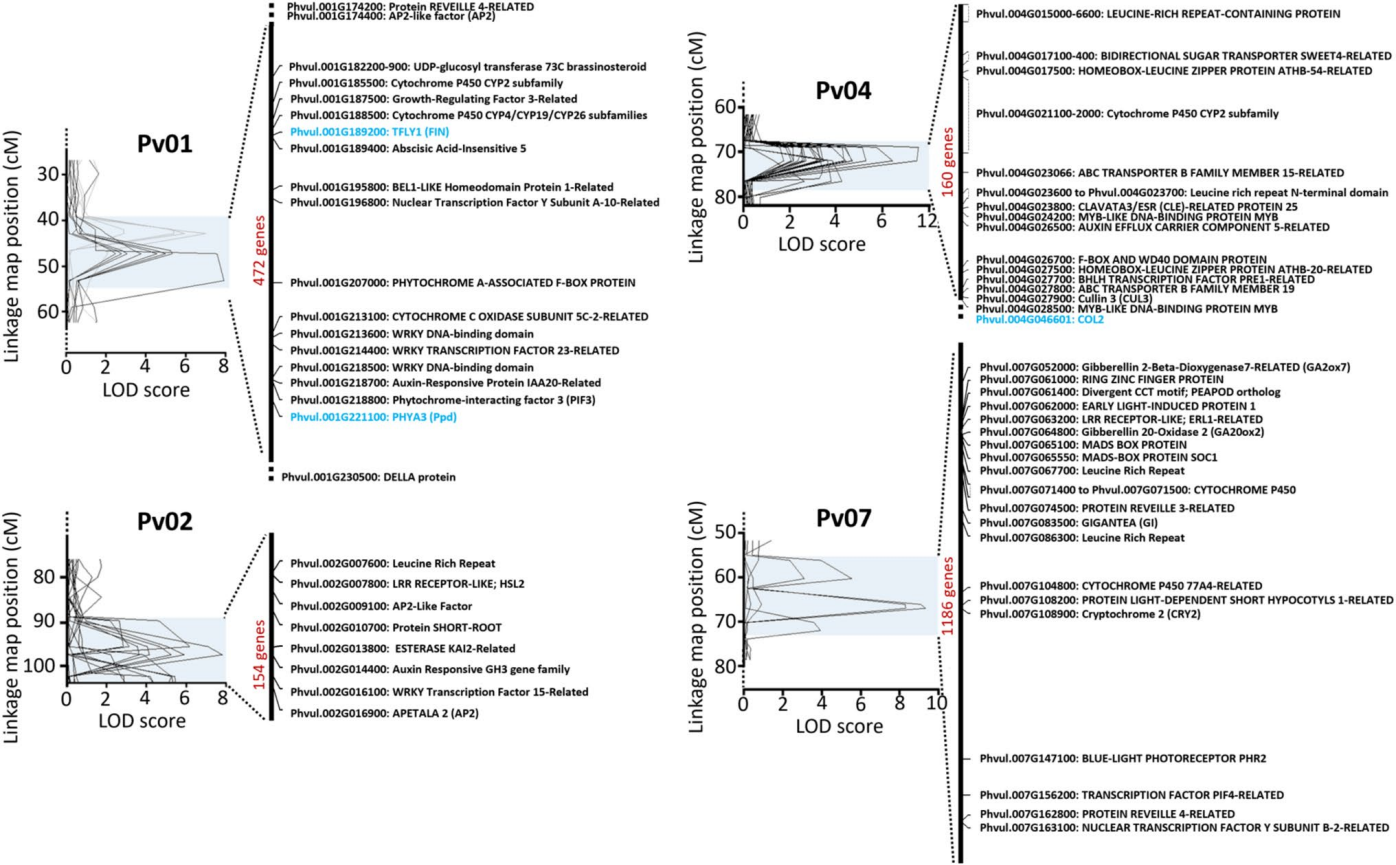
Identified candidate genes for each of the four main internode QTL genomic regions identified in the F_2_ *Phaseolus vulgaris* population derived from a cross between the wild G12873 and the domesticated Midas accessions. Flowering candidate genes are highligthed in blue, and the total gene number within each region is denoted in red

In order to relate QTL to the reference genome, the physical position of map markers was determined in the *Phaseolus vulgaris* v2.1 reference genome (Schmutz et al. 2014). The 2-LOD confidence interval for each of the four internode QTL regions on chromosomes 1, 2, 4 and 7 was examined for potential candidate genes. Several positional candidates were identified based on relevant links to internode elongation literature (Fig. 3; Supplementary Table S10).

Interestingly, the Chr01 and Chr04 QTL regions appearred to co-locate with the three known phenology-related genes, *Fin (TFL1y)* (Repinski et al. 2012) and *PHYTOCHROME A3* (*PHYA3*) (Weller et al. 2019) on Chr01, and *CONSTANS-like 2 (COL2)* (González et al. 2021) on Chr04 (Fig. 3). These three phenology loci were segregating in the F_2_ population and genotyped, with NFI recorded. Notably, several peaks in mean internode length across development coincided with the mean NFI observed in the recessive flowering loci classes, *fin/ppd* (NFI=5.4) and *col2* (NFI=21) (Fig. 1c**, **Fig 2b).

Given this correlation, the position of the QTL regions were further investigated. The closely linked flowering genes *Fin* and *Ppd* were located within the combined 2-LOD zone of the two defined QTL regions on Chr01 (Fig. 3), and thus appeared strong candidates*.* The peak marker for the QTL region affecting earlier internodes (internodes 4-9), at 42.9cM, was located less than 50 genes away from *Fin*, while the second region (affecting later internodes 14-21) was somewhat shifted in comparison, with the prominent peak marker at 47.3cM lying slightly closer to *Ppd* (<130 genes away) than to *Fin* (<200 genes away). The third flowering gene *COL2,* was located <300 genes away from the peak marker for the Chr04 QTL region at 71.8cM, and was therefore also considered as a strong candidate.

### QTL validation: fine mapping and HRM analysis

**Fig. 4:**
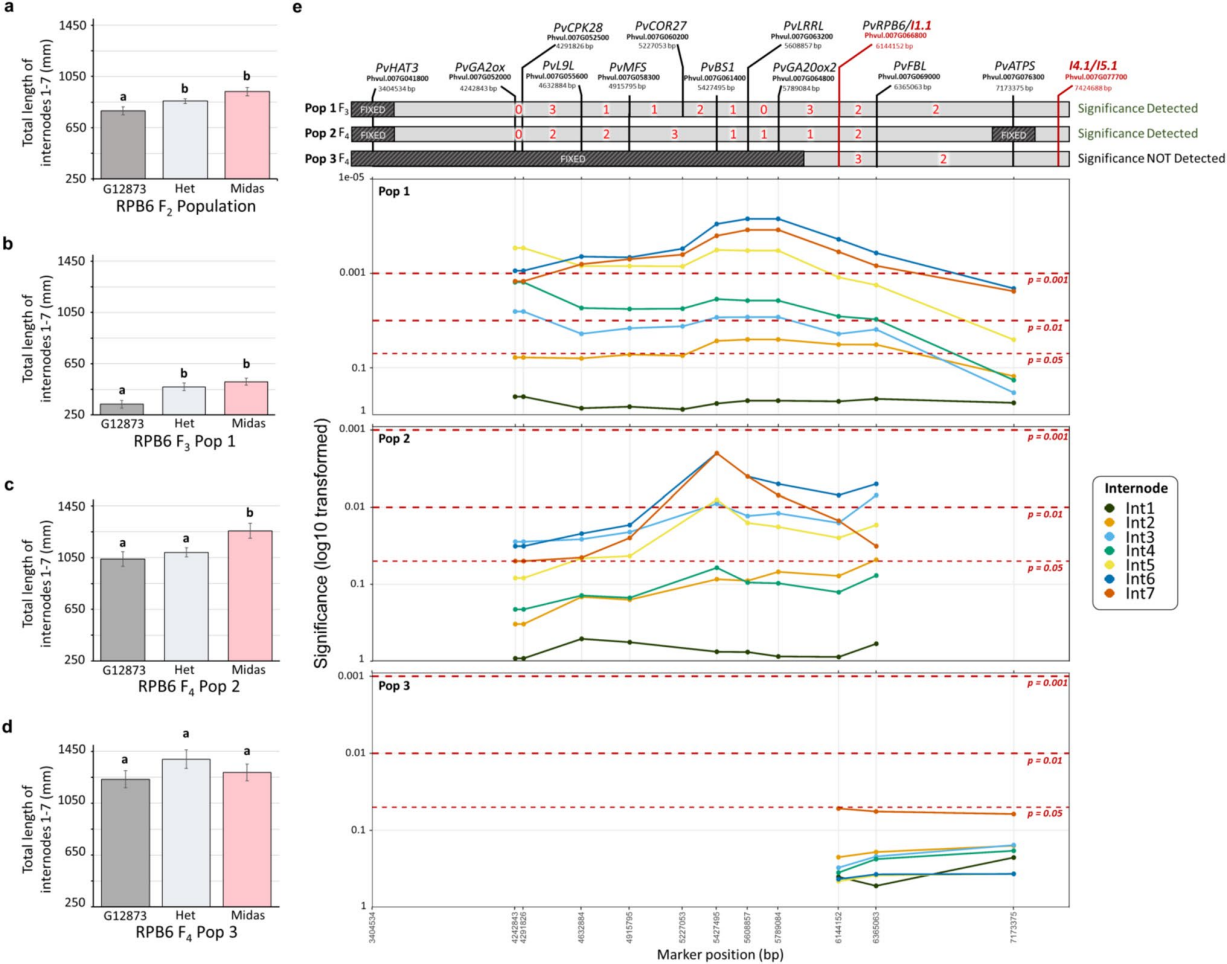
Validation of Chr07 QTL effects in advanced generations. **a-d.** Effect of the genotype at peak marker RPB6 (G12873, Midas or heterozygous) on total length of internodes 1 to 7 in the F_2_ population **a**, an F_3_ progeny (Pop1, n=91) **b**, and two F_4_ progenies (Pop2, *n*=57, and Pop3, *n*=32) **c** and **d**. Error bars represent standard error, and significant differences between means (*P*<0.05) are indicated by different letters. **e** Detailed map and significance of marker effects across the Chr07 QTL region, based on F_3_ (Pop 1) and F_4_ progenies (Pop2 and Pop3). Markers in the map diagram at top are labelled according to annotated gene name, with F_2_ peak markers for individual internode length QTL shown in red, and are ordered based on their positions in the bean reference genome. Red digits denote the number of recombinations identified between indicated pairs of markers, and fixed (non-segregating) regions in each progeny are indicated. The lower panels show the significance of homozygous marker effects on individual internode lengths (Int1-Int7) in each of the three populations, with the dashed lines at P=0.05 representing the significance threshold. Internode QTLs I2.2 and I3.1 were located to a scaffold region of the genome and could not be resolved in this diagram

The two QTL regions with the clearest effect on elongation across early internodes, located on Chr07 and Chr04, were investigated in more detail. In the F_2_ population, the Midas allele at the Chr07 region was associated with significantly longer internodes. For example, the Midas allele had a 19.3% increase in elongation between nodes 0 (cotyledon) and 7 (L0-7) at the peak I1.1 marker RPB6 (Fig. 4a). The effect of this QTL was further tested in an F_3_ and two F_4_ populations.

The F_3_ population (population 1) was fixed for the wild and domesticated genotype at several other loci affecting the length of relevant internodes (1-7) (Supplementary Table S2) and was also fixed for the domesticated genotype in the area of Chr07 above the QTL region (Fig. 4e). To increase marker density around the QTL, additional markers were designed and genotyped in this population. Two F_4_ populations were derived from individuals carrying new recombinations — one (population 2) additionally fixed (wild genotype) for the area below the QTL, and another (population 3) in which the area initially fixed (domesticated allele) in the F_3_ was extended by approximately 2.4 Mb towards the QTL (Fig. 4e). Marker genotypes across the region were associated with a significant increase in elongation across multiple internodes in both population 1 and population 2. For example, the *RBP6* marker was associated with an increase in L0-7 of 52.5% in population 1 (Fig. 4b) and 21.0% in population 2 (Fig. 4c). This confirmed the broad location of the QTL between markers *HAT3* and *ATPS* (Fig. 4e). The lack of effect in population 3 (Fig. 4d) further narrowed its location to the 2.4Mb region between *HAT3* and *GA20OX2*, which contains approximately 230 genes (Fig. 4e).

**Fig. 5:**
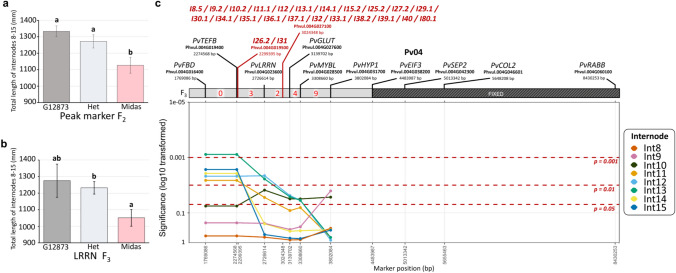
Validation of Chr04 QTL effects in an advanced generation. **a-b**. Effect of the genotype at the peak DArT marker and designed HRM marker LRRN (G12873, Midas or heterozygous) on total length of internodes 8 to 15 in the F_2_ population **a**, and an F_3_ progeny (n=30) respectively **b**. Error bars represent standard error, and significant differences between means (*P*<0.05) are indicated by different letters. **c** Detailed map and significance of marker effects across the Chr04 QTL region, based on the F_3_ progeny. Markers in the map diagram at top are labelled according to annotated gene name, with F_2_ peak markers for individual internode length QTL shown in red, and are ordered based on their positions in the bean reference genome. Red digits denote the number of recombinations identified between indicated pairs of markers, and fixed (non-segregating) regions in each progeny are indicated. The lower panel shows the significance of heterozygote and Midas (domesticated) marker effects on individual internode lengths (Int8-Int15) in the F_3_ progeny, with the dashed lines at *P*=0.05 representing the significance threshold

To investigate the relationship between the Chr04 region affecting later internodes (8-15) and *COL2*, we selected and grew a single F_3_ progeny segregating for the peak markers but fixed for the adjacent region containing *COL2,* the other flowering loci on Chr01 (*Fin* and *Ppd*), and several other loci affecting the length of relevant internodes (Supplementary Table S3). We then scanned the 2-LOD confidence interval of the QTL to identify other potential candidates and designed several additional markers across the region to clarify the extent of the fixed region. In general, the effect of the QTL region in the F_3_ progeny was consistent with its effects in the F_2_. This is illustrated for the total length of internodes 8-15 (Fig. 5a and b) which showed a similar mean decrease in Midas homozygotes relative to the other genotypes, in both the F_2_ (approx. 15%) and the F_3_ (approx. 20%). Notably, extensive variation was observed in F_3_ wild-type individuals, resulting in no statistical differences in internode length compared to Midas homozygotes, while the significant difference to heterozygotes observed in the F_2_ was maintained (Fig. 5a and b). Similar to the Chr07 cluster, marker genotypes across the QTL region were associated with a significant increase in elongation across all internodes excluding internode 8, although the markers associated with internode 9 changes was only shared with internode 10 (Fig. 5c).

Overall, these results support the existence of an elongation QTL distinct from *COL2* in a region of approximately 4.4Mb bounded at one end by the top of chromosome 4 and at the other by the *EIF3* marker. This region contains approximately 380 genes, including several candidates involved in auxin transport (PIN, Phvul.004G026500; ABCB transporters, Phvul.004G023066, Phvul.004G027800) and sugar transport (SWEET4-Related genes, Phvul.004017100–Phvul.004017400). While these results do not rule out contribution from *COL2* to this QTL region initially detected in F_2_, the similar effect size suggests this contribution would be small at best.

### Flowering and elongation

A clear genetic association between flowering time and internode length has been detected in this study, both through close co-location of known phenology genes near QTL regions and the alignment of these regions with time of flowering (Fig. 3; Fig. 2b). For the Chr04 QTL at least, this is unlikely to be a direct effect as it is genetically separable from the adjacent major flowering gene *COL2* **(**Fig. 5b and c**)**. However, surprisingly, although the Midas allele at this QTL was associated with reduced elongation of many internodes in the 10-40 range, this effect was not detected across a narrower zone from nodes 15 to 24 (Fig. 2b), which roughly corresponded to the range in flowering node of *col2* segregants in a functional *Fin/Ppd* background (17-27; mean NFI=21± SD 2.7).

**Fig. 6:**
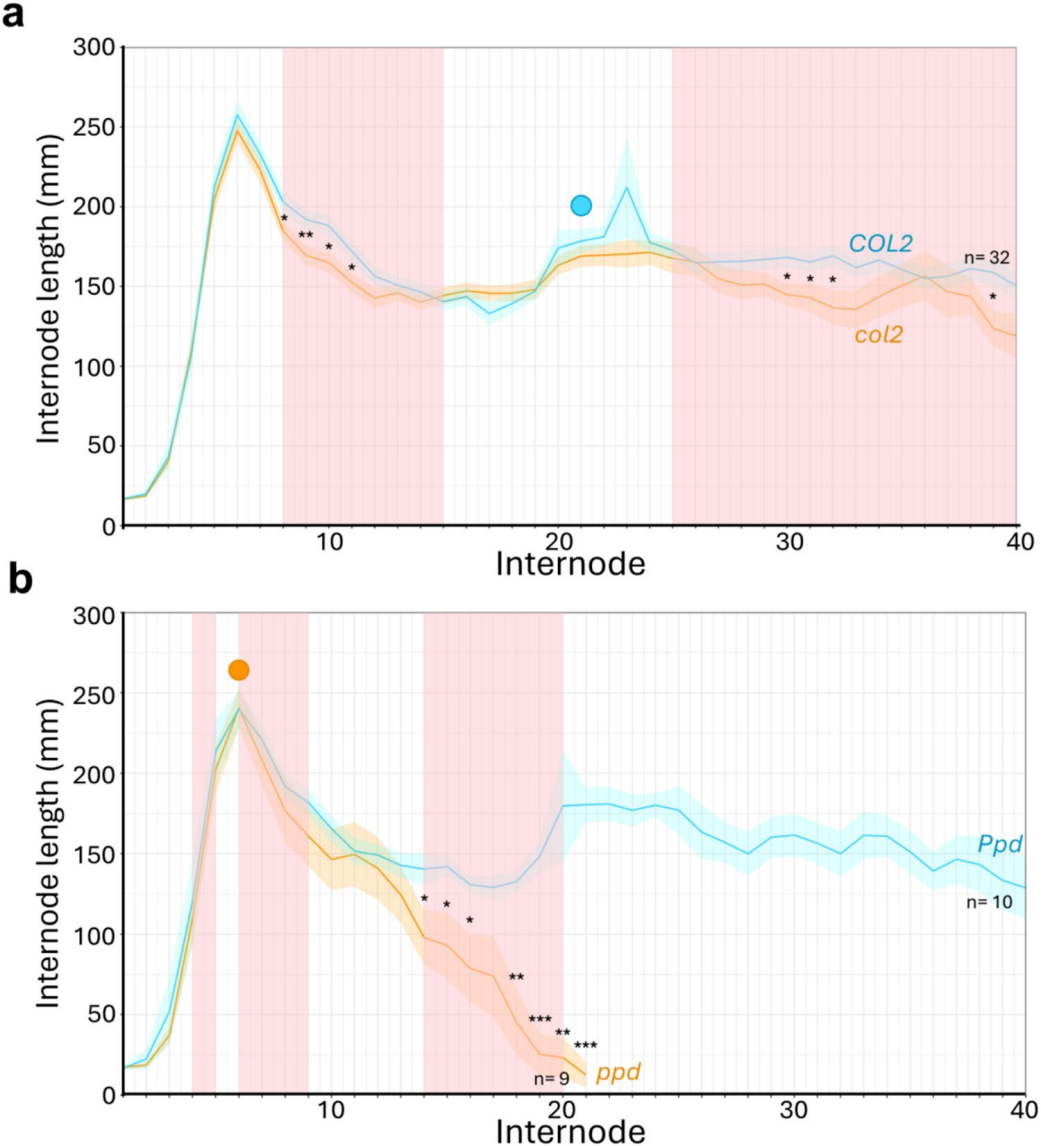
Mean internode length effects of individual flowering time loci in the F_2_ population from a cross between the wild G12873 and the domesticated Midas. **a** Comparison of *COL2* vs *col2* individuals in a *WT Ppd/Fin* background. The internodes for which the Chr04 QTL was detected are indicated by the red shading. Mean NFI for *col2* individuals is denoted by the blue circle. **b** Comparison of *Ppd* vs *ppd* individuals in a WT *COL2* and heterozygous *Fin*/*fin* background. The internodes for which Chr01 QTL were detected are marked by the red shading. Mean NFI for *ppd* individuals is denoted by the orange circle. In both a and b, the standard error for each genotypic class is represented by the corresponding colour shaded area around the mean, and significant differences in internode length between genotypes are denoted by * (* for *P* ≤ 0.05, ** for *P* ≤ 0.01, and *** for *P* ≤ 0.001)

One explanation is that the flowering of *col2* plants could be directly associated with an increase in elongation which might mask the elongation-inhibiting effect of the Midas allele at the adjacent Chr04 QTL over these internodes. This prompted a closer examination of elongation over this region in different genotypes. Fig. 6a shows that when *ppd* and *fin* segregants were excluded from the population, individuals carrying the *col2* mutation exhibited decreased elongation compared to homozygous *COL2* individuals in most of the internodes for which the QTL effect was detected. However, internodes closer to and within the *col2* flowering range (NFI: 17-27) did not exhibit a significant difference (Fig. 6a), suggesting that flowering may be increasing internode length and minimising the difference between genotypes. Similarly, when considering the F_2_ population regardless of flowering genotype, the mean total length of the four internodes spanning the NFI was significantly greater than that of the preceding four internodes (Supplementary Figure S4), further supporting an increase in elongation of internodes surrounding floral initiation.

The apparent co-location of Chr01 elongation QTLs with *fin* and *ppd* suggests these loci might also influence elongation. This region is complicated by linkage between *Fin* and *Ppd*, but there was sufficient recombination to allow the effect of *Ppd* to be examined separately. A comparison of homozygous *ppd* and *Ppd* individuals on a heterozygous *Fin* (indeterminate) background revealed a gradual reduction in elongation over later internodes in *ppd* individuals (Fig. 6b), consistent with the effect of the QTL detected for internodes 14-20 (Fig. 2b, Supplementary Table S9). The second QTL region on Chr01 affecting earlier internodes was more closely collocated with *Fin*, but a similar comparison of homozygous *fin* and *Fin* individuals on a heterozygous *Ppd* was limited by small sample size and failed to show a significant difference at any internode (Supplementary Figure S5).

## Discussion

This study examined the genetic control of internode elongation in an F_2_ population derived from a cross between wild (G12873) and domesticated (Midas) bean. Our results show that the genetic control of elongation has a developmental component, with QTL regions identified which affect specific internode intervals. Interestingly, these distinct zones of QTL influence coincided with windows of flowering in subsets of the population (Fig. 2b), suggesting a close association between elongation and flowering. In fact, two of the elongation QTL regions (Chr01 and Chr04) appeared to co-locate with known phenology genes (Fig. 4).

QTL in the Chr01 region appear to represent two distinct loci, and are close to the known phenology loci *Fin* and *Ppd*. Previous studies using populations segregating for determinacy report a major internode length QTL near *fin*, which explained 16-34% of the observed variation (González et al. 2016; Koinange et al. 1996). While both these studies were based on measurement of a single internode (internode 5), they actually report an inconsistent allelic direction, with the recessive *fin* allele associated with both reduced (González et al. 2016) and increased (Koinange et al. 1996) internode elongation. Unsurprisingly, as determinacy was also segregating in our population study, the discrete group of QTL for internodes 4-9 (located near *Fin*) (Fig. 2) likely corresponds to these previously reported loci. Interestingly allelic direction was also inconsistent among these grouped QTL, with the Midas allele conferring increased elongation in earlier internodes (4-5), while decreasing elongation in later internodes (8-9) (Supplementary Table S9).

The second group of QTL in the Chr01 region affected later internodes (14-21), and was located closer to *Ppd,* likely reflecting the reduced elongation observed post-flowering as *ppd* individuals approach apical arrest (Fig. 6b). An effect of *PHYA* genes through flowering on stem elongation, particularly under long-day conditions, has been shown in both pea and Medicago (Jaudal et al. 2020; Weller et al. 1997). However, previous investigation of the *PHYA3* gene in common bean (*Ppd* locus) demonstrated no clear contribution to photomorphogenesis (Weller et al. 2019). A second *PHYA* gene orthologous to the soybean *PHYA1/PHYA2* (*E4*) homeolog pair is also present (*PvPHYA1*), and it is speculated this may reflect possible subfunctionalization in these proteins with respect to flowering control and photomorphogenesis. Better understanding of the Chr01 loci will likely require better definition of the individual contributions of *Fin* and *Ppd*, to exclude the possibility of a separate elongation locus linked to these flowering loci.

The Chr04 QTL region occurs close to the known phenology gene *COL2*. Although recombination indicates this locus is distinct from *COL2* (Fig. 5c), the strong alignment of the QTL effect with the *col2* flowering window suggests that the effect of this locus becomes obscured when flowering is initiated (Fig. 2b). One plausible explanation is that changes in elongation directly associated with early flowering in *col2* individuals overcomes the reduced elongation conferred by the Chr04 locus. This is corroborated by *col2* mutants exhibiting reduced elongation across development, except during the period of flowering (*col2* functional *Fin/Ppd* background individuals NFI=17-27) (Fig. 6a). Consequently, development of isolines separating *COL2* and the Chr04 locus will likely be required to untangle their effects. The Chr04 locus has been narrowed to a 4.4Mb region, in which several candidates involved in auxin transport and sugar transport are present (Fig. 3; Supplementary Table S10), and may correspond to the previously reported internode QTL, *Int3*, which was observed to explain around 20% of the observed variation (Checa and Blair 2008). We also observed a sizable effect in our population, with this locus consistently explaining >8% of the observed variation for the associated internode (Supplementary Table S8).

In contrast to the previously reported Chr01 and Chr04 loci, the locus on Chr07 appears novel. This locus is also distinct in that it had no association with flowering and exhibited the most prominent effect on early internodes. This locus explained 4.8-11.4% of the observed variation across the first five internodes (Supplementary Table S9) and appears to show dominant inheritance, with the Midas allele conferring significant increases in elongation (Fig. 4a, b, c). Through recombination this locus was narrowed to a 2.4Mb region, containing two gibberellin-related candidate genes, *GA2ox7* (Phvul.007G052000) and *GA20ox2* (Phvul.007G064800) (Fig. 3; Supplementary Table S10), in which a loss and gain-of-function mutation respectively, would be consistent with the inferred role of the locus.

Another appealing candidate within this narrowed region is an ortholog of the two tandemly repeated *Arabidopsis* genes, *PEAPOD1* (*PPD1*) and *PEAPOD2* (*PPD2*), known to regulate hypocotyl elongation in Arabidopsis, in addition to other traits including the size of leaves, siliques and seeds, (Gonzalez et al. 2015; Liu et al. 2020; White 2006; White 2022). Induced mutants of *PEAPOD1/2* orthologs in several legumes show a similar effect on organ size, exhibiting both increased leaf and seed size (Ge et al. 2016; Li et al. 2019; Naito et al. 2017). Additionally, variation in a chickpea ortholog has also been associated with leaf and seed size, and a range of other traits including dry shoot weight (Barmukh et al. 2022; Nguyen et al. 2022). Overall, these results are suggestive of a conserved role for *PEAPOD* orthologs in the control of organ size in legumes and provide a strong case for *PvPEAPOD* as a candidate for the Chr07 locus.

The effect of the Chr02 clusters was detected over early internodes (3-9), spanning the fringes of the Chr07 and start of the Chr04 clusters. The effect was later detected (I24-38) broadly across the same region as the later Chr04 cluster effect. Several candidate genes lie within the Chr02 cluster (Fig. 3; Supplementary Table S10), however *Phvul.002G016900* (*APETALA 2)* is a notable candidate, given the demonstrated importance of *AP2* in internode elongation in barley (Patil et al. 2019).

Application of next-generation sequencing technologies have significantly improved the efficacy of genotyping (Varshney et al. 2009), providing access to genomic information across the diversity of common bean. Although genome-wide association studies (GWAS) have yet to examine elongation specifically, associations for plant height, biomass, canopy height, growth habit and lodging have been detected near the Chr01, 2 and 4 loci identified in this study (Delfini et al. 2021; Dramadri et al. 2021; Hoyos-Villegas et al. 2017; MacQueen et al. 2020; Moghaddam et al. 2016; Wu et al. 2020). While these associations may simply reflect the substantial changes in height conferred by determinacy and phenology, they may also potentially reflect specific effects on internode elongation (i.e. elongation loci). While no relevant height-related associations were detected near the Chr07 locus in these studies, a definitive comparison with our results would require specific phenotyping for internode elongation in these panels, an exercise that would additionally allow the effect and relevance of specific candidate SNPs and genes identified here to be further explored across a wider range of germplasm.

Among the numerous QTL studies in common bean, relatively few have specifically examined elongation, but those that have report several other loci additional to the main 4 QTL regions we have identified in this study. Two of these, on Chr03 and Chr04, had substantial effects (15-28% variation explained) (Checa and Blair 2008) whereas others on Chr06 and Chr05 were relatively minor (González et al. 2016). Although these studies also involved crosses between Mesoamerican and Andean gene pools, they used domesticated parents, and our use of a wild x domesticated cross incorporating wider phenological variation is likely to have obscured the effect of other elongation loci.

Changes in plant architecture have been strongly associated with domestication in common bean (Gepts 2014; Kwak et al. 2012; Smartt 1990), but whether modified elongation has been a consistent part of this is unclear. Our cross is not optimal for addressing this question, as it extends across gene pools, and a more direct approach would involve targeted wild x landrace analyses within each germplasm group. Nonetheless, the effect of the Chr07 locus on seedling elongation is likely to be conspicuous, and could potentially have led to selection during domestication, whether directly for increased elongation, or indirectly through effects on improved vigour, competition and harvestability. The effect of such loci would arguably be more conspicuous in a *fin/*determinate background, as such surveying elongation across *fin* diversity/haplotypes may also prove beneficial.

Stem elongation is a major component of plant architecture and a valuable target for crop improvement, influencing canopy structure, lodging tolerance, planting density, harvestability, and ultimately yield in legumes (Farooq et al. 2019; Kuzbakova et al. 2022; McDonald et al. 2013; Murchie and Burgess 2022; Beagley and Weller 2024; Fang et al. 2024). The practical relevance and potential utility of the Chr04 and Chr07 loci in breeding programs is highlighted by their substantial effects, accounting for a total difference in elongation of ≈20% and ≈50% across their respective internode regions. For example, the broad reduction of elongation conferred by the Chr04 locus may promote a more compact growth habit and minimise lodging risk, while the targeted early elongation effect of the Chr07 locus could improve first pod height without compromising overall plant stature, thereby facilitating mechanized harvesting and crop adaptation.

In summary, our comprehensive QTL analysis has identified four main genomic regions controlling internode elongation, two of which appear to be novel. This has provided new insights into the genetic architecture of this trait and has allowed a deeper understanding of its genetic control over development. We also highlight potential links with flowering, which may have important implications for selection on these traits. While close linkage of a separate elongation locus cannot be entirely ruled out, pleiotropic effects of these flowering loci are a more likely explanation. Although the experiment was conducted under controlled, long-day conditions, the strong response of stem elongation to the environment highlights the importance of evaluating these QTL regions across diverse field conditions and photoperiods to assess their stability. Overall, these results demonstrate how a better understanding of the genetic control and interactions of plant architecture traits will be highly relevant for efforts to improve crop adaptation to local growing environments.

## Supplementary Information

Below is the link to the electronic supplementary material.

Supplementary file1 (DOCX 867 KB)

## Data Availability

The datasets generated, including genotype, phenotype and mapping data are available on the University of Tasmania Research Data Portal, Dataset Key RD-GMILPDFFPDFCB [http://rdp.utas.edu.au/metadata/5ec73656-f69f-4820-b84b-e72cd26bd295]. All other data supporting the findings of this study are available within the paper and within its supplementary data published online.

## References

[CR1] Altschul SF, Madden TL, Schäffer AA, Zhang J, Zhang Z, Miller W, Lipman DJ (1997) Gapped BLAST and PSI-BLAST: a new generation of protein database search programs. Nucleic Acids Res 25(17):3389–3402. 10.1093/nar/25.17.3389 9254694 10.1093/nar/25.17.3389 PMC146917

[CR2] Barmukh R, Roorkiwal M, Dixit GP et al (2022) Characterization of ‘QTL-hotspot’ introgression lines reveals physiological mechanisms and candidate genes associated with drought adaptation in chickpea. J Exp Bot. 10.1093/jxb/erac348 36006832 10.1093/jxb/erac348 PMC9730794

[CR3] Beagley CJ, Weller JL (2024) Genetic control of stem elongation in legume crops and its potential relevance. Crop Sci. 10.1002/csc2.21283

[CR5] Butler JB, Vaillancourt RE, Potts BM, Lee DJ, King GJ, Baten A, Shepherd M, Freeman JS (2017) Comparative genomics of *Eucalyptus* and *Corymbia* reveals low rates of genome structural rearrangement. BMC Genomics 18(1):397. 10.1186/s12864-017-3782-7 28532390 10.1186/s12864-017-3782-7 PMC5441008

[CR6] Checa OE, Blair MW (2008) Mapping QTL for climbing ability and component traits in common bean (*Phaseolus vulgaris* L.). Mol Breed 22(2):201–215. 10.1007/s11032-008-9167-5

[CR7] Chen C (2019) Evaluation of the effect of temperature on a stem elongation model of *Phalaenopsis*. Horticulturae 5(4):76. 10.3390/horticulturae5040076

[CR8] Churchill GA, Doerge RW (1994) Empirical threshold values for quantitative trait mapping. Genetics 138(3):963–971. 10.1093/genetics/138.3.963 7851788 10.1093/genetics/138.3.963 PMC1206241

[CR9] Collard B, Mace E, McPhail M, Wenzl P, Cakir M, Fox G, Poulsen D, Jordan D (2009) How accurate are the marker orders in crop linkage maps generated from large marker datasets? Crop Pasture Sci 60(4):362–372. 10.1071/CP08099

[CR10] Delfini J, Moda-Cirino V, dos Santos Neto J, Zeffa DM, Nogueira AF, Ribeiro LAB, Ruas PM, Gepts P, Gonçalves LSA (2021) Genome-Wide Association Study Identifies Genomic Regions for Important Morpho-Agronomic Traits in Mesoamerican Common Bean. Front Plant Sci. 10.3389/fpls.2021.748829 34691125 10.3389/fpls.2021.748829 PMC8528967

[CR11] Doyle JJ, Doyle JL (1987) A rapid DNA isolation procedure for small quantities of fresh leaf tissue. Phytochem Bullet 7:9

[CR12] Dramadri IO, Nkalubo ST, Kramer DM, Kelly JD (2021) Genome-wide association analysis of drought adaptive traits in common bean. Crop Science 61(5):3232–3253. 10.1002/csc2.20484

[CR13] Fang C, Du H, Wang L, Liu B, Kong F (2024) Mechanisms underlying key agronomic traits and implications for molecular breeding in soybean. J Genet Genomics 51(4):379–393. 10.1016/j.jgg.2023.09.004 37717820 10.1016/j.jgg.2023.09.004

[CR14] Farooq M, Hussain M, Ul-Allah S, Siddique KHM (2019) Physiological and agronomic approaches for improving water-use efficiency in crop plants. Agric Water Manage 219:95–108. 10.1016/j.agwat.2019.04.010

[CR15] Ge L, Yu J, Wang H, Luth D, Bai G, Wang K, Chen R (2016) Increasing seed size and quality by manipulating *BIG SEEDS1* in legume species. Proc Natl Acad Sci U S A 113(44):12414–12419. 10.1073/pnas.1611763113 27791139 10.1073/pnas.1611763113 PMC5098654

[CR16] Gepts P (2014) The contribution of genetic and genomic approaches to plant domestication studies. Curr Opin Plant Biol 18:51–59. 10.1016/j.pbi.2014.02.001 24631844 10.1016/j.pbi.2014.02.001

[CR17] Gepts, P., & Debouck, D. (1991). Origin, domestication, and evolution of the common bean. *Common beans: research for crop improvement*, 7-53. Retrieved from https://books.google.com.co/books?id=CvcbUopfa54C&lpg=PA7&ots=ghwJ2Aq-K7&lr&hl=es&pg=PA7#v=onepage&q&f=false

[CR18] González AM, Vander Schoor JK, Fang C, Kong F, Wu J, Weller JL, Santalla M (2021) Ancient relaxation of an obligate short-day requirement in common bean through loss of CONSTANS-like gene function. Current Biol 31(8):1643-1652.e1642. 10.1016/j.cub.2021.01.075 10.1016/j.cub.2021.01.075 33609454

[CR19] González AM, Yuste-Lisbona FJ, Saburido S, Bretones S, De Ron AM, Lozano R, Santalla M (2016) Major contribution of flowering time and vegetative growth to plant production in common bean as deduced from a comparative genetic mapping. Front Plant Sci. 10.3389/fpls.2016.01940 28082996 10.3389/fpls.2016.01940 PMC5183638

[CR20] Gonzalez N, Pauwels L, Baekelandt A et al (2015) A repressor protein complex regulates leaf growth in Arabidopsis. Plant Cell 27(8):2273–2287. 10.1105/tpc.15.00006 26232487 10.1105/tpc.15.00006 PMC4568497

[CR21] Gu W, Zhu J, Wallace DH, Singh SP, Weeden NF (1998) Analysis of genes controlling photoperiod sensitivity in common bean using DNA markers. Euphytica 102(1):125–132. 10.1023/A:1018340514388

[CR22] Haley CS, Knott SA (1992) A simple regression method for mapping quantitative trait loci in line crosses using flanking markers. Heredity 69(4):315–324. 10.1038/hdy.1992.131 16718932 10.1038/hdy.1992.131

[CR23] Hayat I, Asif A, Tariq M, Anwaar A, Bashir S (2014) Nutritional and health perspectives of beans (*Phaseolus vulgaris* L.): an overview. Crit Rev Food Sci Nutr 54(5):580–592 24261533 10.1080/10408398.2011.596639

[CR24] Hedden P (2003) The genes of the green revolution. Trends Genet 19(1):5–9. 10.1016/S0168-9525(02)00009-4 12493241 10.1016/s0168-9525(02)00009-4

[CR25] Hoyos-Villegas V, Song Q, Kelly JD (2017) Genome-wide Association Analysis for Drought Tolerance and Associated Traits in Common Bean. *The Plant Genome, 10*(1), plantgenome2015.2012.0122. 10.3835/plantgenome2015.12.0122 10.3835/plantgenome2015.12.0122 28464065

[CR26] Jaccoud D, Peng K, Feinstein D, Kilian A (2001) Diversity arrays: a solid state technology for sequence information independent genotyping. Nucleic Acids Res 29(4):e25–e25. 10.1093/nar/29.4.e25 11160945 10.1093/nar/29.4.e25 PMC29632

[CR27] Jaudal M, Wen J, Mysore KS, Putterill J (2020) *Medicago* PHYA promotes flowering, primary stem elongation and expression of flowering time genes in long days. BMC Plant Biol 20(1):329. 10.1186/s12870-020-02540-y 32652925 10.1186/s12870-020-02540-y PMC7353751

[CR28] Jighly A, Joukhadar R, Alagu M (2015) SimpleMap: A Pipeline to Streamline High‐Density Linkage Map Construction. *The Plant Genome, 8*(2), plantgenome2014.2009.0056. 10.3835/plantgenome2014.09.0056 10.3835/plantgenome2014.09.0056 33228304

[CR29] Kelly JD (2001) Remaking bean plant architecture for efficient production. Adv Agronomy 71(71):109–143. 10.1016/s0065-2113(01)71013-9

[CR30] Koinange EMK, Singh SP, Gepts P (1996) Genetic control of the domestication syndrome in common bean. Crop Sci 36:4

[CR31] Kosambi DD (1943) The estimation of map distances from recombination values. Ann Eugen 12(1):172–175. 10.1111/j.1469-1809.1943.tb02321.x

[CR32] Kuzbakova M, Khassanova G, Oshergina I et al (2022) Height to first pod: a review of genetic and breeding approaches to improve combine harvesting in legume crops. Front Plant Sci. 10.3389/fpls.2022.948099 36186054 10.3389/fpls.2022.948099 PMC9523450

[CR33] Kwak M, Toro O, Debouck DG, Gepts P (2012) Multiple origins of the determinate growth habit in domesticated common bean (*Phaseolus vulgaris*). Ann Bot 110(8):1573–1580. 10.1093/aob/mcs207 23019270 10.1093/aob/mcs207 PMC3503494

[CR34] Kwak M, Velasco D, Gepts P (2008) Mapping homologous sequences for determinacy and photoperiod sensitivity in common bean (*Phaseolus vulgaris*). J Hered 99(3):283–291. 10.1093/jhered/esn005 18316323 10.1093/jhered/esn005

[CR35] Lester DR, Ross JJ, Davies PJ, Reid JB (1997) Mendel’s stem length gene (Le) encodes a gibberellin 3 beta-hydroxylase. Plant Cell 9(8):1435–1443. 10.1105/tpc.9.8.1435 9286112 10.1105/tpc.9.8.1435 PMC157009

[CR36] Li X, Liu W, Zhuang LL et al (2019) Bigger organs and elephant ear-like *LEAF1* control organ size and floral organ internal asymmetry in pea. J Exp Bot 70(1):179–191. 10.1093/jxb/ery352 30295864 10.1093/jxb/ery352

[CR37] Lisciani S, Marconi S, Le Donne C et al (2024) Legumes and common beans in sustainable diets: nutritional quality, environmental benefits, spread and use in food preparations. Front Nutr 11:1385232. 10.3389/fnut.2024.1385232 38769988 10.3389/fnut.2024.1385232 PMC11104268

[CR38] Liu Z, Li N, Zhang Y, Li Y (2020) Transcriptional repression of GIF1 by the KIX-PPD-MYC repressor complex controls seed size in Arabidopsis. Nat Commun 11(1):1846. 10.1038/s41467-020-15603-3 32296056 10.1038/s41467-020-15603-3 PMC7160150

[CR39] MacQueen AH, White JW, Lee R, Osorno JM, Schmutz J, Miklas PN, Myers J, McClean PE, Juenger TE (2020) Genetic associations in four decades of multienvironment trials reveal agronomic trait evolution in common bean. Genetics 215(1):267–284. 10.1534/genetics.120.303038 32205398 10.1534/genetics.120.303038 PMC7198278

[CR40] Martinez O, Curnow R (1992) Estimating the locations and the sizes of the effects of quantitative trait loci using flanking markers. Theor Appl Genet 85(4):480–488. 10.1007/bf00222330 24197463 10.1007/BF00222330

[CR41] McDonald MR, Gossen BD, Kora C, Parker M, Boland G (2013) Using crop canopy modification to manage plant diseases. Eur J Plant Pathol 135:581–593. 10.1007/s10658-012-0133-z

[CR42] Moghaddam SM, Mamidi S, Osorno JM* et al* (2016) Genome-Wide Association Study Identifies Candidate Loci Underlying Agronomic Traits in a Middle American Diversity Panel of Common Bean. *The Plant Genome, 9*(3), plantgenome2016.2002.0012. 10.3835/plantgenome2016.02.0012 10.3835/plantgenome2016.02.0012 27902795

[CR43] Murchie EH, Burgess AJ (2022) Casting light on the architecture of crop yield. Crop Environ 1(1):74–85. 10.1016/j.crope.2022.03.009

[CR44] Naito K, Takahashi Y, Chaitieng B et al (2017) Multiple organ gigantism caused by mutation in *VmPPD* gene in blackgram (*Vigna mungo*). Breed Sci 67(2):151–158. 10.1270/jsbbs.16184 28588392 10.1270/jsbbs.16184 PMC5445970

[CR45] Nguyen DT, Hayes JE, Harris J, Sutton T (2022) Fine mapping of a vigor QTL in chickpea (*Cicer arietinum* L.) reveals a potential role for Ca4_TIFY4B in regulating leaf and seed size. Front Plant Sci. 10.3389/fpls.2022.829566 35283931 10.3389/fpls.2022.829566 PMC8908238

[CR46] Nyholt DR (2000) All LODs are not created equal. Am J Hum Genet 67(2):282–288. 10.1086/303029 10884360 10.1086/303029 PMC1287176

[CR47] Ohtaka K, Yoshida A, Kakei Y, Fukui K, Kojima M, Takebayashi Y, Yano K, Imanishi S, Sakakibara H (2020) Difference between day and night temperatures affects stem elongation in tomato (*Solanum lycopersicum*) seedlings via regulation of gibberellin and auxin synthesis. Front Plant Sci. 10.3389/fpls.2020.577235 33363551 10.3389/fpls.2020.577235 PMC7752778

[CR48] Patil V, McDermott HI, McAllister T et al (2019) APETALA2 control of barley internode elongation. Development. 10.1242/dev.170373 31076487 10.1242/dev.170373 PMC6589076

[CR49] Peng J, Richards DE, Hartley NM et al (1999) ‘Green revolution’ genes encode mutant gibberellin response modulators. Nature 400(6741):256–261. 10.1038/22307 10421366 10.1038/22307

[CR50] Repinski SL, Kwak M, Gepts P (2012) The common bean growth habit gene PvTFL1y is a functional homolog of Arabidopsis TFL1. Theor Appl Genet 124(8):1539–1547. 10.1007/s00122-012-1808-8 22331140 10.1007/s00122-012-1808-8

[CR51] Rieu I, Ruiz-Rivero O, Fernandez-Garcia N et al (2008) The gibberellin biosynthetic genes *AtGA20ox1* and *AtGA20ox2* act, partially redundantly, to promote growth and development throughout the Arabidopsis life cycle. Plant J 53(3):488–504. 10.1111/j.1365-313X.2007.03356.x 18069939 10.1111/j.1365-313X.2007.03356.x

[CR52] Ronin Y, Mester D, Minkov D, Korol A (2010) Building reliable genetic maps: different mapping strategies may result in different maps. Nat Sci 2(6):576–589. 10.4236/ns.2010.26073

[CR53] Sansaloni C, Petroli C, Jaccoud D, Carling J, Detering F, Grattapaglia D, Kilian A (2011) Diversity arrays technology (DArT) and next-generation sequencing combined: genome-wide, high throughput, highly informative genotyping for molecular breeding of Eucalyptus. BMC Proc 5(7):P54. 10.1186/1753-6561-5-s7-p5422373051

[CR54] Schmutz J, McClean PE, Mamidi S et al (2014) A reference genome for common bean and genome-wide analysis of dual domestications. Nat Genet 46(7):707–713. 10.1038/ng.3008 24908249 10.1038/ng.3008 PMC7048698

[CR55] Smartt J (1990) *Grain legumes: evolution and genetic resources*: Cambridge university press.

[CR56] Spielmeyer W, Ellis MH, Chandler PM (2002) Semidwarf (sd-1), “green revolution” rice, contains a defective gibberellin 20-oxidase gene. Proc Natl Acad Sci U S A 99(13):9043–9048. 10.1073/pnas.132266399 12077303 10.1073/pnas.132266399 PMC124420

[CR57] Sturtevant A (1913) The linear arrangement of six sex-linked factors in *Drosophila* as shown by mode of association. J Exp Zool 14:39–45. 10.1002/jez.1400140104

[CR58] Toro O, Tohme J, Debouck D (1990) Wild bean (Phaseolus vulgaris L.): description and distribution. *181*. Retrieved from https://books.google.com.co/books?id=uHdaJmba3CMC&pg=PP3&dq=Wild+bean+(Phaseolus+vulgaris+L.):+Description+and+distribution.#v=onepage&q=&f=false

[CR59] van den Ende H, Zeevaart JAD (1971) Influence of daylength on gibberellin metabolism and stem growth in *Silene armeria*. Planta 98(2):164–176. 10.1007/BF00385349 24493350 10.1007/BF00385349

[CR60] Van Ooijen J (2006) JoinMap® 4, Software for the calculation of genetic linkage maps in experimental populations. *Kyazma BV, Wageningen, 33*(10.1371).

[CR61] Van Ooijen J (2009) MapQTL® 6, Software for the mapping of quantitative trait loci in experimental populations of diploid species. *Kyazma BV, Wageningen, Netherlands*.

[CR62] Varshney RK, Nayak SN, May GD, Jackson SA (2009) Next-generation sequencing technologies and their implications for crop genetics and breeding. Trends Biotechnol 27(9):522–530. 10.1016/j.tibtech.2009.05.006 19679362 10.1016/j.tibtech.2009.05.006

[CR63] Voorrips R (2002) MapChart: software for the graphical presentation of linkage maps and QTLs. J Hered 93(1):77–78. 10.1093/jhered/93.1.77 12011185 10.1093/jhered/93.1.77

[CR64] Wallace DH, Yourstone KS, Masaya PN, Zobel RW (1993) Photoperiod gene control over partitioning between reproductive and vegetative growth. Theor Appl Genet 86(1):6–16. 10.1007/bf00223803 24193378 10.1007/BF00223803

[CR65] Wang B, Smith SM, Li J (2018) Genetic regulation of shoot architecture. Annu Rev Plant Biol 69:437–468. 10.1146/annurev-arplant-042817-040422 29553800 10.1146/annurev-arplant-042817-040422

[CR66] Weller JL, Murfet IC, Reid JB (1997) Pea mutants with reduced sensitivity to far-red light define an important role for phytochrome a in day-length detection. Plant Physiol 114(4):1225–1236. 10.1104/pp.114.4.1225 12223768 10.1104/pp.114.4.1225 PMC158415

[CR67] Weller JL, Vander Schoor JK, Perez-Wright EC, Hecht V, González AM, Capel C, Yuste-Lisbona FJ, Lozano R, Santalla M (2019) Parallel origins of photoperiod adaptation following dual domestications of common bean. J Exp Bot 70(4):1209–1219. 10.1093/jxb/ery455 31222352 10.1093/jxb/ery455

[CR68] White DW (2006) Peapod regulates lamina size and curvature in Arabidopsis. Proc Natl Acad Sci USA 103(35):13238–13243. 10.1073/pnas.0604349103 16916932 10.1073/pnas.0604349103 PMC1550771

[CR69] White DWR (2022) Peapod repressors modulate and coordinate developmental responses to light intensity in Arabidopsis. New Phytol 235(4):1470–1485. 10.1111/nph.18198 35510737 10.1111/nph.18198 PMC9544094

[CR70] Wu J, Wang LF, Fu JJ et al (2020) Resequencing of 683 common bean genotypes identifies yield component trait associations across a north-south cline. Nat Genet 52(1):118. 10.1038/s41588-019-0546-0 31873299 10.1038/s41588-019-0546-0

[CR71] Wu W, Chen L, Liang R et al (2025) The role of light in regulating plant growth, development and sugar metabolism: a review. Front Plant Sci. 10.3389/fpls.2024.1507628 39840366 10.3389/fpls.2024.1507628 PMC11747448

