# Supplementary material for: Genetic control of stem elongation in common bean and the influence of age and flowering time
Source: Theor Appl Genet. 2025 Aug 21;138(9):222. doi: 10.1007/s00122-025-04996-8 (PMC12370798; doi:10.1007/s00122-025-04996-8)
Supplement: Supplementary file 1 — Supplementary file1 (DOCX 867 KB) [file 122_2025_4996_MOESM1_ESM.docx]

***Genetic control of stem elongation in common bean and the influence of age and flowering time***

Chantelle J. Beagley^1,2^, Jacqueline K. Vander Schoor^1,2^, Jakob B. Butler^1,2^, and James L. Weller^1,2^*

^1^School of Natural Sciences, University of Tasmania, Private Bag 55, Hobart, TAS 7001, Australia

^2^ARC Centre of Excellence for Plant Success in Nature and Agriculture, University of Tasmania, Hobart, TAS 7001, Australia

*Corresponding author

Chantelle J. Beagley: [chantelle.beagley@utas.edu.au](mailto:chantelle.beagley@utas.edu.au), ORCID: 0000-0002-4254-0510

Jacqueline K. Vander Schoor: [jacqueline.vanderschoor@utas.edu.au](mailto:jacqueline.vanderschoor@utas.edu.au), ORCID: 0000-0002-3813-5678

Jakob B. Butler: [jakob.butler@utas.edu.au](mailto:jakob.butler@utas.edu.au), ORCID: 0000-0003-1353-3765

James L. Weller: [jim.weller@utas.edu.au](mailto:jim.weller@utas.edu.au), ORCID: 0000-0003-2423-8286

# Supplementary Figures


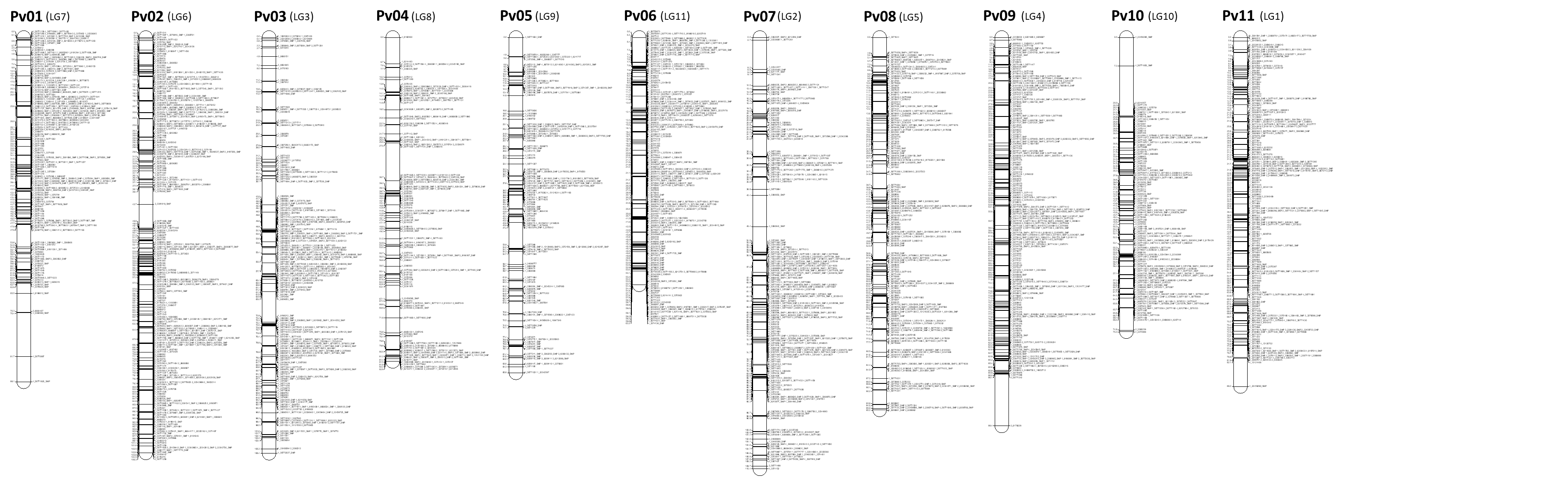


**Fig. S1.** Finalised G12873 x Midas linkage map consisting of 3286 markers. Linkage groups (LG) arranged in order of the chromosome they correspond to. Figure created in MapChart v2.2 (Voorrips, 2002).


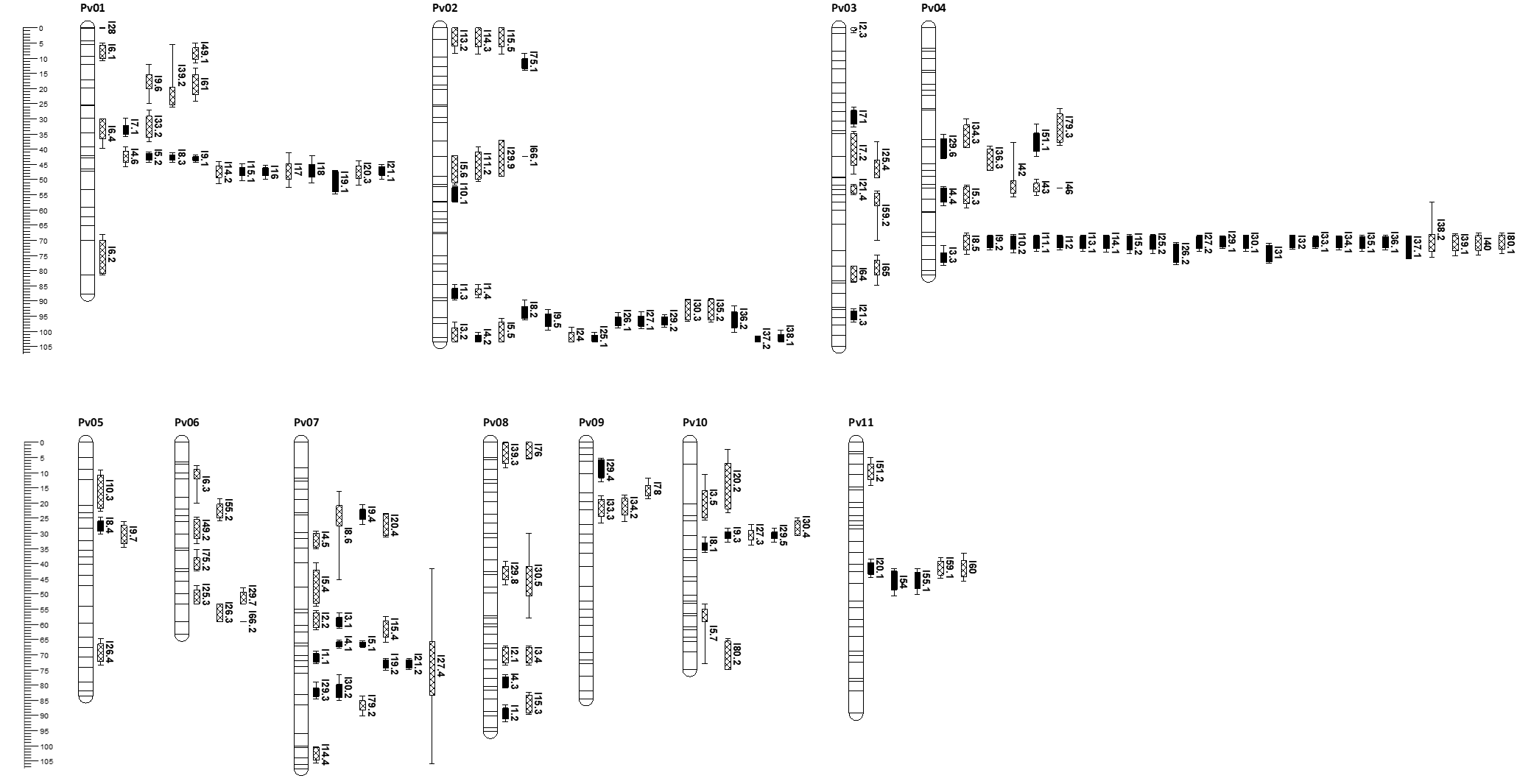


**Fig. S2.** Location of internode length QTL on the 11 Phaseolus vulgaris skeleton linkage maps. QTL are displayed to the right of each linkage group, with their respective internode number and relative LOD score. Significant (genome-wide) level QTL are presented with solid bars, while suggestive (chromosome-wide) level QTL are specified by hatched bars. One-LOD and two-LOD confidence intervals are denoted by the bars and lines respectively. Horizontal lines on the linkage groups display marker positions. Figure created in MapChart v2.2 (Voorrips, 2002).


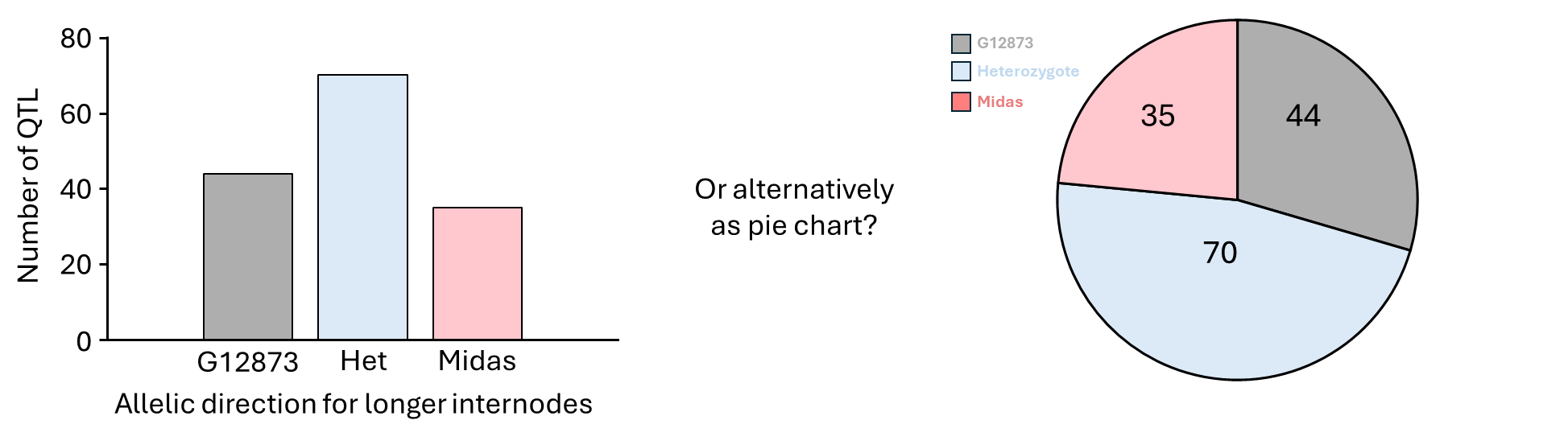


**Fig. S3.** Allelic direction of all (n=149) internode length QTL identified in a common bean F_2_ population derived from a cross between a wild (G12873) and domesticated (Midas) line. Longer internodes were conferred by wild alleles (G12873 and Heterozygote) more frequently (77%), compared to domesticated (Midas) alleles (23%).


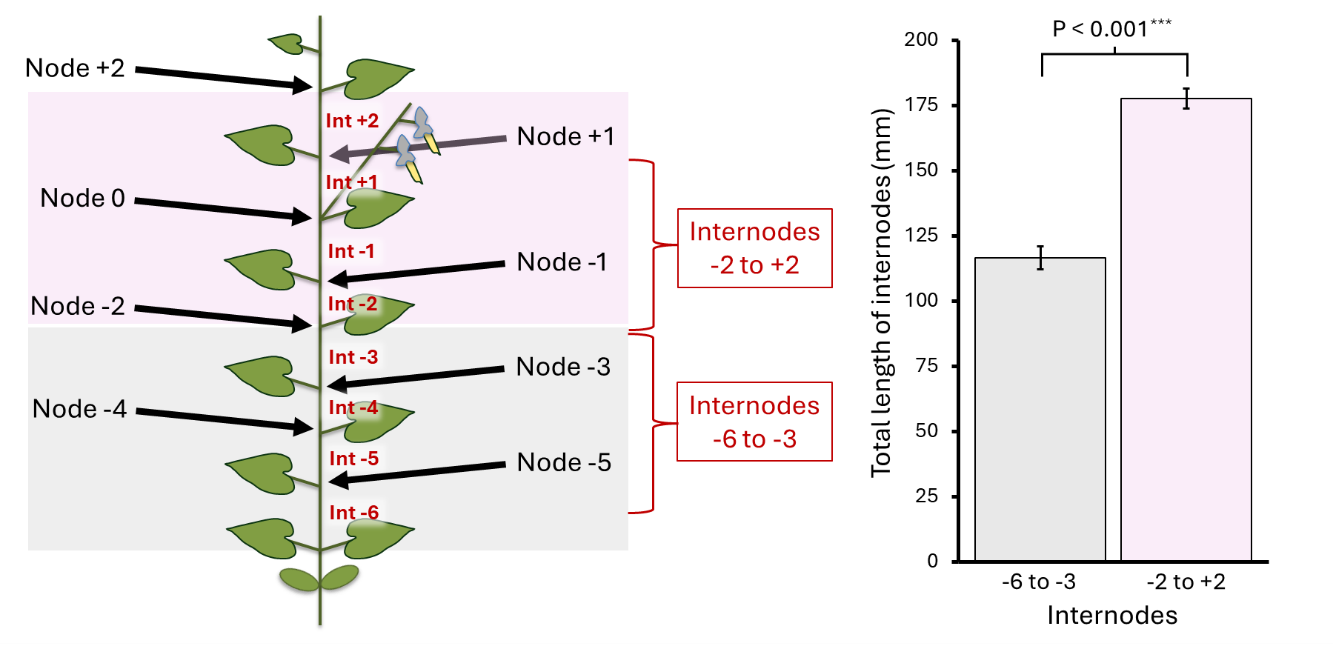


**Fig. S4.** Comparison of mean internode length across the F_2_ population in the two internodes surrounding the NFI (Int -2 to +2) against the four internodes prior to flowering (Int -6 to -3). Significant difference between these two groups was observed, with P < 0.001***.


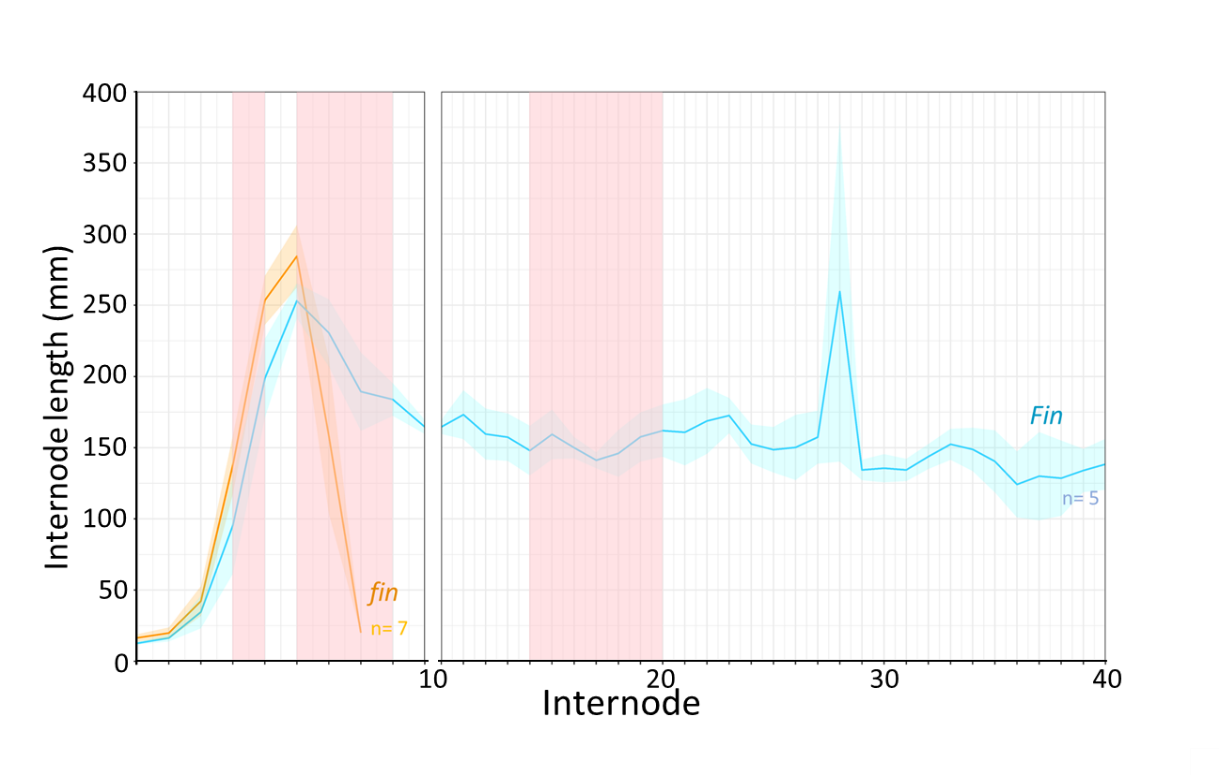


**Fig. S5.** Comparison of mean internode length in *Fin* vs *fin* individuals in a *Ppd* heterozygous and *COL2* WT background. Standard error for each genotypic class is represented by the corresponding colour shaded area, the internodes for which Chr01 loci QTL were detected is marked by the red shading. No significant difference in internode length was detected between genotypes for any internode.

# **Supplementary Tables**

**Table S1.** Criteria used to rank a.) dominant (presence or absence) and b.) co-dominant (one dose, two doses or no doses) DArT markers before linkage map construction.

| **a.)** | **Rank** | **Depth** | **Call Rate (%)** | **Polymorphism information content (PIC)** | **Reproducibility (%)** |
| --- | --- | --- | --- | --- | --- |
|  | 1 | ≥13 | ≥0.90 | ≥0.38 | 1 |
|  | 2 | ≥10 | ≥0.89 | ≥0.30 | 1 |
|  | 3 | ≥10 | ≥0.8 | ≥0.15 | ≥0.973 |
|  | 4 | ≥5 | ≥0.8 | ≥0.15 | ≥0.95 |

| **b.)** | **Rank** | **Avg Count SNP** | **Call Rate (%)** | **Avg Polymorphism information content (PIC)** | **Reproducibility Avg (%)** |
| --- | --- | --- | --- | --- | --- |
|  | 1 | ≥10 | ≥1 | 0.36 | 1 |
|  | 2 | ≥8 | ≥1 | 0.33 | ≥0.98 |
|  | 3 | ≥5 | ≥1 | 0.3 | ≥0.98 |
|  | 4 | ≥3 | ≥0.8 | 0.25 | ≥0.9 |

**Table S2.** The genotypes of the F_2_ individual chosen to be grown on from for the F_3_ internode Chr07 QTL cluster follow up. Several QTL for Internodes 1-5 at other loci were fixed, this individual was also fixed WT for the flowering and maturity loci *Fin, Ppd* and *COL2* using the HRM markers outlined in Supplementary Table 4*.*

| **Selected F_2_ individual for F_3_ generation** | | | |
| --- | --- | --- | --- |
| **Fixed Wild allele** | **Heterozygous** | **Fixed Domesticated allele** | **Unsure of genotype** |
| I4.4 | **I1.1** | I2.1 | I1.2 |
| I5.2 | **I2.2** | I3.4 | I2.3 |
| I5.3 | **I3.1** | I4.3 |  |
| I3.5 | **I4.1** | I4.5 |  |
| I4.6 | **I5.1** |  |  |
| *Fin* | i1.3 |  |  |
| *Ppd* | i1.4 |  |  |
| *COL2* | i3.2 |  |  |
|  | i3.3 |  |  |
|  | i4.2 |  |  |
|  | I5.5 |  |  |
|  | I5.4 |  |  |
|  | I5.6 |  |  |
|  | I5.7 |  |  |
|  |  |  |  |
|  | **QTL we are following up** | |  |
|  | **QTL we are trying to fix** | |  |

**Table S3.** The genotypes of the F_2_ individual chosen to be grown on from for the F_3_ internode Chr04 QTL cluster follow up. Several QTL for Internodes 8-15 at other loci were fixed, this individual was also fixed WT for the flowering and maturity loci *Fin, Ppd* and *COL2.*

| **Selected F_2_ individual for F_3_ generation** | | | |
| --- | --- | --- | --- |
| **Fixed Wild allele** | **Heterozygous** | **Fixed Domesticated allele** | **Unsure of genotype** |
| I8.4 | I8.5 | I8.6 | I11.2 |
| I9.6 | I9.2 | I9.4 |  |
| I14.2 | I10.2 | I10.1 |  |
| I15.1 | I11.1 | I15.4 |  |
| I9.7 | I12 |  |  |
| *Fin* | I13.1 |  |  |
| *Ppd* | I14.1 |  |  |
| *COL2* | I15.2 |  |  |
|  | I8.1 |  |  |
|  | I8.2 |  |  |
|  | I8.3 |  |  |
|  | I9.1 |  |  |
|  | I9.3 |  |  |
|  | I9.5 |  |  |
|  | I10.3 |  |  |
|  | I13.2 |  |  |
|  | I14.3 |  |  |
|  | I14.4 |  |  |
|  | I15.3 |  |  |
|  | I15.5 |  |  |
|  |  |  |  |
|  | **QTL we are following up** | |  |
|  | **QTL we are trying to fix** | |  |

**Table S4.** Details of primer pairs used for genotyping flowering loci in the F_2_ population derived from G12873 X Midas cross.

| **Gene** | **ID** | **Marker name** | **Forward and reverse sequence** | **Tm (°C)** | **Candidate gene for** |
| --- | --- | --- | --- | --- | --- |
| PvTFLY1 | Phvul.001G189200 | PvTFL1y-F12  PvTFL1y-R11 | F: CAAACCAACAGTAAAAACCAG | 58 | Genotyping for Fin |
|  |  |  | R: TTGTAGGATCGTTGTGTCG |  |  |
| PvPHYAb | Phvul.001G221100 | PvPHYAb-5F  PvPHYAb-5R | F: TGTGGCCCAAGATATAACTGC | 58 | Genotyping for Ppd |
|  |  |  | R: GAGGGATCAATGGGTTTCG |  |  |
| PvRPL9 | Phvul.004G046700 | PvRPL9-2F  PvRPL9-2R | F: GAGAATCTGATACCTTCTATACCAA | 59 | Genotyping for COL2 |
|  |  |  | R: CAAAATCACACTTTGTAAGACACAA |  |  |

**Table S5.** Details of primer pairs used for HRM genotyping in segregating Chr07 and Chr04 internode populations.

| **Gene** | **ID** | **Marker name** | **Forward and reverse sequence** | **Tm (°C)** | **Candidate gene for** |
| --- | --- | --- | --- | --- | --- |
| PvHAT3 | Phvul.007G041800 | PvHAT3 2F/2R | F: CCCGAGTATGATTGGTAGTTGC | 59 | Chr07 QTL cluster |
|  |  |  | R: GAATACACATTTCTAGCATTTGAAGG |  |  |
| PvGA20ox3 | Phvul.007G052000 | PvGA20ox3-1F/1R | F: ATTCAAACCCACCCCTTCAC | 58 | Chr07 QTL cluster |
|  |  |  | R: TCGTTACTGTTTCGGCTCTTG |  |  |
| PvCPK28 | Phvul.007G052500 | PvCPK28 3F/3R | F: TTTCAGGGTAGGCTCTCCAA | 58 | Chr07 QTL cluster |
|  |  |  | R: TCTGACAACCTTTTGACTGC |  |  |
| PvL9L | Phvul.007G055600 | PvL9L-2F/2R | F: GAAGACATTCCTGGACATGGTC | 59 | Chr07 QTL cluster |
|  |  |  | R: GGGTATAACGATGAGCAGGGT |  |  |
| PvMFS | Phvul.007G058300 | PvMFS-1F/1R | F: TCTTCTTTGCCACCTTGATTCT | 57 | Chr07 QTL cluster |
|  |  |  | R: CGATGATGAATTTCCATGGATCA |  |  |
| PvCOR27 | Phvul.007G060200 | PvCOR27-3F/3R | F: GCCTAACACATGACCAGTACAG | 58 | Chr07 QTL cluster |
|  |  |  | R: GGTGTATGAGTAGCTACCAAGAT |  |  |
| PvBS1 | Phvul.007G061400 | PvBS1 6F/6R | F: AAATAAATCAGATTGTCCTGTTTGC | 59 | Chr07 QTL cluster |
|  |  |  | R: CCCCCTGCTTGTTAGATGG |  |  |
| PvLRRL | Phvul.007G063200 | PvLRRL-2F/2R | F: TGAGTACCGCAATTCCTTTTGT | 58 | Chr07 QTL cluster |
|  |  |  | R: ACGTTACAATGCAGAAGACATGT |  |  |
| PvGA20ox2 | Phvul.007G064800 | PvGA20ox2-3F/3R | F: GCGATAAAATGGGCAAAGAGT | 57 | Chr07 QTL cluster |
|  |  |  | R: CACATGTGTTTGATTACCCGAAT |  |  |
| PvRPB6 | Phvul.007G066800 | PvRPB6 2F/2R | F: TTCAGTCTCTACCTGCACAATATC | 57 | Chr07 QTL cluster |
|  |  |  | R: AGGTTATAATGTAGGGCCTGAAG |  |  |
| PvFBL | Phvul.007G069000 | PvFBL-2F/2R | F: ACTTTCCCTTGGTGCCTAAAAC | 58 | Chr07 QTL cluster |
|  |  |  | R: TCCCAAGAGCTACTCCTGATAAA |  |  |
| PvATPS | Phvul.007G076300 | PvATPS 3F/3R | F: AAATCGATGAGAAGATGCAA | 56 | Chr07 QTL cluster |
|  |  |  | R: TTGTGGGGATAACAGAGTCA |  |  |
| PvRABB | Phvul.004G060100 | PvRABB 4F/4R | F: TCATTTCTATGTGGTTCTGGAAAG | 60 | Chr04 QTL cluster |
|  |  |  | R: CTTGGTGTGTCTTTGGTTTCAC |  |  |
| PvCOL2 | Phvul.004G046700 | PvRPL9 2F/2R | F: GAGAATCTGATACCTTCTATACCAA | 59 | Chr04 QTL cluster |
|  |  |  | R: CAAAATCACACTTTGTAAGACACAA |  |  |
| PvSEP2 | Phvul.004G042300 | PvSEP2 3F/3R | F: CCGTTGTTAATTATAGGATGAAGG | 59 | Chr04 QTL cluster |
|  |  |  | R: TGCTCTGCTACAAAAGCAAATG |  |  |
| PvEIF3 | Phvul.004G038200 | PvEIF3 3F/3R | F: CCCATGGTAAATGATATCTCTAAA | 58 | Chr04 QTL cluster |
|  |  |  | R: GCAACATATATCACACGACACCA |  |  |
| PvHYP1 | Phvul.004G031700 | PvHYP1 3F/3R | F: TGAATTCATTGTCCAAGACTACAAA | 58 | Chr04 QTL cluster |
|  |  |  | R: CATTAAGACAGATTTTTCGCATA |  |  |
| PvFBD | Phvul.004G016400 | PvFBD 1F/1R | F: TCATAGCATCACGTAACAAAGG | 55 | Chr04 QTL cluster |
|  |  |  | R: TTTACGAACCTTGAAATTTTGC |  |  |
| PvTEFB | Phvul.004G019400 | PvTEFB 1F/1R | F: CCAAATCAACAAAGACGAATCC | 57 | Chr04 QTL cluster |
|  |  |  | R: TGAACGGAAGTTGTTGTTGG |  |  |
| PvLRRN | Phvul.004G023600 | PvLRRN 1F/1R | F: CCTTCTTTATACTCGCTTAAACTTCC | 58 | Chr04 QTL cluster |
|  |  |  | R: TGACACTGCGTGTGTTTGG |  |  |
| PvGLUT | Phvul.004G027600 | PvGLUT 2F/2R | F: AAGGCCGTGTTGATGATCC | 57 | Chr04 QTL cluster |
|  |  |  | R: TTGCCATTATCAACGAAACC |  |  |
| PvMYBL | Phvul.004G028500 | PvMYBL 1F/1R | F: TGAGATGTATCACAAGTGACAACTG | 57 | Chr04 QTL cluster |
|  |  |  | R: TGGTGATAGATTCTGAGTAATTCTTG |  |  |

**Table S6.** Mean internode length, sample size and standard error for internode length measurements in an F_2_ population derived from a G12873 (wild) x Midas (Domesticated) cross. Mean internode length (mm) is coloured from largest (Red) to smallest (Green) values.

| **Internode** | **0-1** | **2** | **3** | **4** | **5** | **6** | **7** | **8** | **9** | **10** | **11** | **12** | **13** | **14** | **15** | **16** | **17** | **18** | **19** | **20** |
| --- | --- | --- | --- | --- | --- | --- | --- | --- | --- | --- | --- | --- | --- | --- | --- | --- | --- | --- | --- | --- |
| **Mean (mm)** | 16.5 | 21.3 | 50.0 | 133.0 | 227.1 | 243.7 | 217.9 | 189.4 | 177.9 | 172.1 | 161.8 | 154.2 | 149.8 | 143.8 | 143.8 | 141.0 | 137.6 | 140.7 | 146.8 | 165.8 |
| **Number of Individuals** | 264 | 264 | 264 | 263 | 263 | 240 | 212 | 193 | 191 | 189 | 189 | 189 | 187 | 186 | 186 | 186 | 183 | 181 | 181 | 178 |
| **Standard Error** | 0.26 | 0.85 | 2.42 | 3.99 | 3.68 | 3.59 | 3.83 | 3.33 | 2.79 | 2.86 | 2.76 | 2.69 | 2.55 | 2.79 | 2.71 | 2.82 | 3.11 | 3.13 | 3.31 | 5.85 |
|  |  |  |  |  |  |  |  |  |  |  |  |  |  |  |  |  |  |  |  |  |
| **Internode** | **21** | **22** | **23** | **24** | **25** | **26** | **27** | **28** | **29** | **30** | **31** | **32** | **33** | **34** | **35** | **36** | **37** | **38** | **39** | **40** |
| **Mean (mm)** | 170.0 | 173.0 | 178.5 | 173.1 | 171.3 | 166.8 | 162.2 | 162.6 | 161.7 | 158.4 | 157.2 | 155.0 | 155.4 | 156.0 | 154.6 | 153.3 | 153.6 | 152.6 | 148.0 | 148.1 |
| **Number of Individuals** | 176 | 173 | 173 | 173 | 173 | 172 | 171 | 170 | 166 | 165 | 162 | 162 | 160 | 155 | 151 | 148 | 146 | 142 | 140 | 138 |
| **Standard Error** | 3.44 | 2.89 | 6.63 | 3.23 | 3.41 | 3.44 | 3.17 | 4.76 | 3.03 | 3.21 | 3.27 | 3.41 | 3.75 | 3.91 | 3.82 | 3.74 | 4.23 | 3.84 | 3.96 | 4.59 |
|  |  |  |  |  |  |  |  |  |  |  |  |  |  |  |  |  |  |  |  |  |
| **Internode** | **41** | **42** | **43** | **44** | **45** | **46** | **47** | **48** | **49** | **50** | **51** | **52** | **53** | **54** | **55** | **56** | **57** | **58** | **59** | **60** |
| **Mean (mm)** | 147.3 | 150.7 | 151.7 | 147.9 | 150.3 | 145.4 | 148.5 | 143.6 | 148.6 | 147.9 | 153.5 | 151.2 | 150.7 | 145.2 | 147.1 | 148.7 | 153.5 | 148.8 | 152.9 | 155.9 |
| **Number of Individuals** | 135 | 128 | 125 | 123 | 120 | 116 | 110 | 105 | 101 | 99 | 95 | 94 | 94 | 93 | 89 | 88 | 81 | 78 | 78 | 75 |
| **Standard Error** | 4.68 | 4.57 | 5.01 | 5.16 | 5.46 | 5.69 | 5.28 | 5.50 | 5.32 | 5.65 | 5.66 | 5.32 | 5.77 | 6.12 | 6.23 | 6.04 | 6.43 | 5.79 | 5.85 | 6.63 |
|  |  |  |  |  |  |  |  |  |  |  |  |  |  |  |  |  |  |  |  |  |
| **Internode** | **61** | **62** | **63** | **64** | **65** | **66** | **67** | **68** | **69** | **70** | **71** | **72** | **73** | **74** | **75** | **76** | **77** | **78** | **79** | **80** |
| **Mean (mm)** | 148.3 | 143.0 | 144.8 | 143.1 | 138.1 | 145.1 | 157.0 | 155.8 | 160.2 | 158.8 | 164.4 | 155.0 | 153.6 | 149.8 | 142.8 | 140.7 | 128.2 | 131.5 | 122.8 | 97.2 |
| **Number of Individuals** | 71 | 69 | 65 | 60 | 60 | 54 | 48 | 46 | 44 | 41 | 37 | 36 | 34 | 30 | 28 | 26 | 25 | 21 | 20 | 18 |
| **Standard Error** | 7.06 | 7.10 | 8.39 | 8.09 | 8.77 | 8.48 | 8.79 | 9.33 | 8.37 | 9.17 | 7.92 | 7.79 | 8.57 | 7.66 | 10.00 | 10.88 | 10.37 | 13.15 | 13.33 | 14.23 |
|  |  |  |  |  |  |  |  |  |  |  |  |  |  |  |  |  |  |  |  |  |
| **Internode** | **81** | **82** | **83** | **84** | **85** | **86** | **87** | **88** | **89** | **90** | **91** | **92** |  |  |  |  |  |  |  |  |
| **Mean (mm)** | 94.6 | 89.2 | 75.1 | 59.7 | 68.6 | 87.5 | 95.0 | 94.3 | 64.0 | 53.5 | 43.3 | 25.0 |  |  |  |  |  |  |  |  |
| **Number of Individuals** | 15 | 13 | 7 | 6 | 5 | 4 | 4 | 4 | 4 | 4 | 3 | 2 |  |  |  |  |  |  |  |  |
| **Standard Error** | 13.23 | 11.83 | 9.54 | 17.92 | 21.15 | 16.42 | 20.26 | 26.12 | 20.23 | 21.35 | 18.78 | 5.00 |  |  |  |  |  |  |  |  |

**Table S7.** Summary table of the constructed finalised linkage map statistics for an F_2_ population derived from a G12873 (wild) x Midas (domesticated) cross*.*

| **Chromosome** | **Markers Mapped to Correct Chromosome** | **Markers Mapped to Scaffold** | **Markers Incorrectly Mapped to Chromosome** | **Markers Not Mapped to the Reference Genome** |
| --- | --- | --- | --- | --- |
| **Pv01** | 247 | 0 | 14 | 26 |
| **Pv02** | 329 | 7 | 13 | 57 |
| **Pv03** | 292 | 1 | 13 | 45 |
| **Pv04** | 183 | 1 | 6 | 36 |
| **Pv05** | 165 | 13 | 10 | 12 |
| **Pv06** | 277 | 3 | 6 | 31 |
| **Pv07** | 295 | 9 | 12 | 40 |
| **Pv08** | 276 | 0 | 10 | 34 |
| **Pv09** | 265 | 1 | 19 | 29 |
| **Pv10** | 176 | 3 | 10 | 33 |
| **Pv11** | 218 | 4 | 22 | 43 |
| **Total** | 2723 | 42 | 135 | 386 |

**Table S8.** QTL analysis results for the length of each internode. Table details the map position, peak QTL marker, genome position in the common bean reference genome (specified where markers blasted to a different chromosome than mapped to), LOD score, percentage of variation explained by the QTL (PVE), * signifies where a LOD score was significant (above genome wide significance), and estimated average values for the genotypes G12873 (mu_A), Midas (mu_B) and the HETs (mu_H) of identified QTL. Allelic direction for longer internodes is specified in the last column.

| **QTL** | **Chromosome** | **CentiMorgan** | **Associated Marker** | **Genome Position (bp)** | **LOD** | **PVE (%)** | **mu_A** | **mu_H** | **mu_B** | ***Allelic direction*** |
| --- | --- | --- | --- | --- | --- | --- | --- | --- | --- | --- |
| I1.1 | 7 | 72 | 1_56771959 | 6145270 | 3.98* | 5.8 | 15.1604 | 16.0239 | 17.9867 | B |
| I1.2 | 8 | 90.1 | 2_56770981 | 782272 | 3.96* | 5.8 | 18.0295 | 16.5762 | 15.1177 | A |
| I1.3 | 2 | 88.9 | 1_56771558 | 2133560 | 3.21 | 4.6 | 16.2773 | 18.4051 | 16.8699 | H |
| I1.4 | 2 | 57.4 | 1_3367050 | 29602765 | 3.19 | 4.6 | 15.3615 | 15.368 | 17.7857 | B |
| I2.1 | 8 | 71.7 | 1_8178332 | 3659035 | 3.43 | 5.3 | 23.4628 | 16.9042 | 15.1645 | A |
| I2.2 | 7 | 60.3 | 1_56770837 | scaffold_16 | 3.14 | 4.8 | 14.9238 | 18.9116 | 23.7035 | B |
| I2.3 | 3 | 0 | 1_8213988 | 367259 | 2.68 | 4.1 | 20.5606 | 25.2843 | 18.0667 | H |
| I3.1 | 7 | 60.3 | 1_56770837 | scaffold_16 | 5.59* | 7.6 | 19.4133 | 32.5408 | 50.6057 | B |
| I3.2 | 2 | 102.1 | 1_22342965 | 547948 | 5.28* | 7.2 | 39.2385 | 56.433 | 30.7805 | H |
| I3.3 | 4 | 76.4 | 1_8212370 | 2304527 | 3.98* | 5.3 | 36.5792 | 55.0503 | 33.4398 | H |
| I3.4 | 8 | 71.7 | 1_8178332 | 3659035 | 3.41 | 4.6 | 46.3412 | 30.3617 | 23.6777 | A |
| I3.5 | 10 | 24.2 | 1_56771008 | 43016914 | 2.66 | 3.5 | 24.8449 | 30.5693 | 45.1741 | B |
| I4.1 | 7 | 67 | 1_56771207 | 7430931 | 8.36* | 10.5 | 83.5435 | 128.945 | 149.616 | B |
| I4.2 | 2 | 103.4 | 1_22459442 | 520142 | 5.47* | 6.7 | 123.752 | 151.437 | 109.408 | H |
| I4.3 | 8 | 80.5 | 1_8208657 | 2299272 | 4.61* | 5.6 | 137.06 | 105.11 | 96.1002 | A |
| I4.4 | 4 | 56.5 | 1_56771424 | Chr11: 7490510 | 4.36* | 5.3 | 118.537 | 148.229 | 114.623 | H |
| I4.5 | 7 | 31.6 | 1_3369616 | 36180793 | 3.48 | 4.2 | 124.825 | 89.8472 | 108.335 | A |
| I4.6 | 1 | 42.9 | 1_3370781 | 45286620 | 2.93 | 3.5 | 100.56 | 106.275 | 132.6 | B |
| I5.1 | 7 | 67 | 1_56771207 | 7430931 | 9.38* | 11.4 | 179.779 | 222.146 | 253.627 | B |
| I5.2 | 1 | 42.9 | 1_3370781 | 45286620 | 5.43* | 6.4 | 197.948 | 201.231 | 235.457 | B |
| I5.3 | 4 | 56.5 | 1_56771423 | 5288363 | 3.19 | 3.7 | 224.812 | 240.347 | 208.594 | H |
| I5.4 | 7 | 47.7 | 4_3380842_SNP | 30773068 | 3.14 | 3.6 | 235.545 | 210.986 | 197.861 | A |
| I5.5 | 2 | 102.1 | 1_22342965 | 547948 | 2.73 | 3.1 | 222.362 | 237.714 | 211.043 | H |
| I5.6 | 2 | 48.9 | 1_56771485 | 35006255 | 2.61 | 3 | 233.395 | 223.181 | 200.01 | A |
| I5.7 | 10 | 57.1 | 1_3367261 | Chr05: 36200850 | 2.34 | 2.7 | 219.059 | 196.118 | 214.347 | A |
| I6.1 | 1 | 9.4 | 1_3374599 | Chr02: 42127879 | 3.58 | 5.2 | 233.16 | 226.11 | 193.057 | A |
| I6.2 | 1 | 70.1 | 4_3380580_SNP | 49809393 | 3.1 | 4.5 | 219.422 | 235.4 | 206.795 | H |
| I6.3 | 6 | 12 | 1_3366348 | 18738726 | 2.54 | 3.7 | 223.533 | 229.717 | 202.684 | H |
| I6.4 | 1 | 34.5 | 1_56771237 | 41207455 | 2.51 | 3.6 | 233.98 | 219.4 | 192.237 | A |
| I7.1 | 1 | 34.5 | 1_56771237 | 41207455 | 7.37* | 14.1 | 230.907 | 222.642 | 160.943 | A |
| I7.2 | 3 | 42.4 | 4_3368529_SNP | 8004098 | 2.7 | 4.9 | 181.924 | 205.313 | 209.925 | B |
| I8.3 | 1 | 42.9 | 1_3370781 | 45286620 | 5.96* | 9 | 183.16 | 169.889 | 101.457 | A |
| I8.2 | 2 | 95.5 | 1_8664417 | 1131098 | 6.2* | 9.4 | 160.473 | 160.752 | 124.143 | H |
| I8.4 | 5 | 28.4 | 1_3366875 | 36088642 | 4.41* | 6.6 | 145.242 | 117.933 | 139.367 | A |
| I8.1 | 10 | 35.3 | 1_56770942 | 41054908 | 6.51* | 9.9 | 127.296 | 166.257 | 157.32 | H |
| I8.5 | 4 | 71.8 | 4_56771900 | 3026374 | 3.28 | 4.8 | 155.776 | 156.356 | 128.84 | H |
| I8.6 | 7 | 23.9 | 1_22342286 | 37358460 | 3.14 | 4.6 | 156.318 | 141.558 | 128.298 | A |
| I9.1 | 1 | 42.9 | 1_3370781 | 45286620 | 7.04* | 10.1 | 172.12 | 159.679 | 87.493 | A |
| I9.5 | 2 | 97.3 | 1_8181646 | 829245 | 4.16* | 5.8 | 142.142 | 140.697 | 117.471 | A |
| I9.2 | 4 | 71.8 | 4_56771900 | 3026374 | 7.04* | 10.1 | 146.884 | 144.529 | 112.728 | A |
| I9.4 | 7 | 23.9 | 1_22342286 | 37358460 | 4.23* | 5.9 | 141.947 | 121.842 | 117.665 | A |
| I9.3 | 10 | 30.8 | 1_56770958 | 41898336 | 4.93* | 6.9 | 116.044 | 138.079 | 143.568 | B |
| I9.6 | 1 | 17.2 | 1_56771221 | 6451683 | 2.99 | 4.1 | 141.656 | 142.593 | 117.957 | H |
| I9.7 | 5 | 32.4 | 1_56771357 | 34659657 | 2.87 | 3.9 | 128.136 | 112.31 | 131.442 | B |
| I10.1 | 2 | 57.2 | 2_22345488 | 27745276 | 5.04* | 10.2 | 184.373 | 176.906 | 150.458 | A |
| I10.2 | 4 | 71.8 | 4_56771900 | 3026374 | 3.98* | 8 | 182.839 | 178.83 | 151.992 | A |
| I10.3 | 5 | 20.9 | 1_3369752 | 37855972 | 2.64 | 5.2 | 172.657 | 151.002 | 162.174 | A |
| I11.1 | 4 | 71.8 | 4_56771900 | 3026374 | 5.19* | 11.1 | 166.928 | 161.84 | 131.758 | A |
| I11.2 | 2 | 48.9 | 1_56771485 | 35006255 | 3.27 | 6.8 | 164.598 | 154.908 | 134.088 | A |
| I12 | 4 | 71.8 | 4_56771900 | 3026374 | 6.53* | 14.7 | 157.689 | 165.02 | 125.598 | H |
| I13.1 | 4 | 71.8 | 4_56771900 | 3026374 | 4.8* | 10.6 | 151.226 | 161.24 | 129.152 | H |
| I13.2 | 2 | 3.7 | 1_22342438 | 49148458 | 2.82 | 6.1 | 151.83 | 141.78 | 128.548 | A |
| I14.1 | 4 | 71.8 | 4_56771900 | 3026374 | 4.8* | 9.4 | 120.905 | 126.516 | 93.582 | H |
| I14.2 | 1 | 47.3 | 1_3378797 | 46538386 | 3.09 | 5.9 | 129.506 | 132.23 | 84.9816 | H |
| I14.3 | 2 | 3.7 | 1_22342438 | 49148458 | 2.75 | 5.3 | 119.898 | 106.64 | 94.5898 | A |
| I14.4 | 7 | 104 | 2_8668406 | 363485 | 2.69 | 5.2 | 99.0543 | 121.415 | 115.433 | H |
| I15.1 | 1 | 47.3 | 1_3378797 | 46538386 | 4.57* | 8.4 | 146.107 | 149.872 | 94.8513 | H |
| I15.2 | 4 | 71.8 | 4_56771900 | 3026374 | 4.09* | 7.4 | 133.205 | 135.553 | 107.753 | H |
| I15.3 | 8 | 88.6 | 1_56771370 | 1224944 | 3.07 | 5.5 | 112.022 | 106.304 | 128.936 | B |
| I15.4 | 7 | 62.5 | 1_56771023 | 8896706 | 2.94 | 5.3 | 108.532 | 115.053 | 132.426 | B |
| I15.5 | 2 | 3.7 | 1_22342438 | 49148458 | 2.85 | 5.1 | 131.399 | 123.417 | 109.56 | A |
| I16 | 1 | 47.3 | 1_3378797 | 46538386 | 5.01* | 11.7 | 145.562 | 143.608 | 78.9387 | A |
| I17 | 1 | 47.3 | 1_3378797 | 46538386 | 2.51 | 6.1 | 143.982 | 137.601 | 83.0019 | A |
| I18 | 1 | 47.3 | 1_3378797 | 46538386 | 3.79* | 9.2 | 149.186 | 139.808 | 69.2402 | A |
| I19.1 | 1 | 53.2 | 1_3380906 | 47899383 | 7.93* | 16.5 | 163.115 | 153.877 | 89.1504 | A |
| I19.2 | 7 | 73.9 | 1_56770963 | 4850687 | 4.59* | 9.1 | 107.74 | 115.473 | 144.526 | B |
| I20.1 | 11 | 42.6 | 4_3381244 | 5756845 | 4.2* | 8.8 | 143.679 | 112.425 | 200.052 | B |
| I20.2 | 10 | 20.4 | 1_3370704 | 43424725 | 2.87 | 5.9 | 184.424 | 136.218 | 159.011 | A |
| I20.3 | 1 | 47.3 | 1_3378797 | 46538386 | 2.8 | 5.8 | 236.728 | 217.979 | 106.706 | A |
| I20.4 | 7 | 29.8 | 1_56771007 | 35593415 | 2.5 | 5.1 | 149.808 | 155.13 | 193.627 | B |
| I21.1 | 1 | 47.3 | 1_3378797 | 46538386 | 5.35* | 10.4 | 173.528 | 170.78 | 48.8917 | A |
| I21.2 | 7 | 73.9 | 1_56770963 | 4850687 | 4.45* | 8.6 | 91.9651 | 105.625 | 130.455 | B |
| I21.3 | 3 | 95.6 | 1_56771138 | 52282729 | 3.88* | 7.4 | 127.844 | 99.0875 | 94.576 | A |
| I21.4 | 3 | 53.2 | 2_8203122 | 35492994 | 2.77 | 5.2 | 109.509 | 132.751 | 112.911 | H |
| I24 | 2 | 103.4 | 1_22459442 | 520142 | 2.89 | 7.4 | 178.12 | 181.626 | 152.348 | H |
| I25.1 | 2 | 103.4 | 1_22459442 | 520142 | 5.35* | 10.9 | 146.334 | 154.428 | 114.951 | H |
| I25.2 | 4 | 71.8 | 4_56771900 | 3026374 | 3.85* | 7.7 | 145.635 | 151.425 | 115.65 | H |
| I25.3 | 6 | 53.2 | 2_8209582 | 29742716 | 2.92 | 5.8 | 121.158 | 147.301 | 140.127 | H |
| I25.4 | 3 | 49.2 | 1_56771473 | 32827655 | 2.53 | 5 | 130.859 | 151.815 | 130.426 | H |
| I26.1 | 2 | 97.3 | 1_8181646 | 829245 | 5.92* | 11.6 | 173.483 | 176.275 | 137.556 | H |
| I26.2 | 4 | 76.4 | 1_8212370 | 2304527 | 5.71* | 11.2 | 173.681 | 181.167 | 137.358 | H |
| I26.3 | 6 | 59 | 1_3369356 | 30724075 | 3.4 | 6.4 | 146.582 | 174.322 | 164.457 | H |
| I26.4 | 5 | 70.8 | 1_3367423 | No hits. | 2.96 | 5.6 | 139.169 | 115.194 | 171.87 | B |
| I27.1 | 2 | 97.3 | 1_8181646 | 829245 | 5.26* | 10.3 | 149.61 | 152.903 | 119.522 | H |
| I27.2 | 4 | 71.8 | 4_56771900 | 3026374 | 4.9* | 9.5 | 151.401 | 152.805 | 117.731 | H |
| I27.3 | 10 | 30.8 | 1_56770958 | 41898336 | 3.2 | 6.1 | 127.585 | 153.39 | 141.547 | H |
| I27.4 | 7 | 72 | 1_56771959 | 6145270 | 2.62 | 4.9 | 121.549 | 129.803 | 147.583 | B |
| I28 | 1 | 0 | 1_8215001 | 1650065 | 3.11* | 8.1 | 193.745 | 154.703 | 146.967 | A |
| I29.1 | 4 | 71.8 | 4_56771900 | 3026374 | 11.23* | 15 | 149.399 | 149.1 | 104.916 | A |
| I29.2 | 2 | 97.3 | 1_8181646 | 829245 | 7.84* | 10 | 141.227 | 145.791 | 113.088 | H |
| I29.3 | 7 | 83 | 4_8201938 | 3419851 | 7.27* | 9.2 | 108.865 | 122.318 | 145.45 | B |
| I29.4 | 9 | 10.3 | 1_56771447 | 36978782 | 4.64* | 5.7 | 110.886 | 119.244 | 143.428 | B |
| I29.5 | 10 | 30.8 | 1_56770958 | 41898336 | 4.34* | 5.3 | 124.726 | 145.564 | 129.589 | H |
| I29.6 | 4 | 39.2 | 2_56771074 | 15682230 | 4.17* | 5 | 117.747 | 109.681 | 136.568 | B |
| I29.7 | 6 | 53.2 | 2_8209582 | 29742716 | 3.53 | 4.2 | 120.562 | 141.21 | 133.753 | H |
| I29.8 | 8 | 43.5 | 2_56770831 | 54012080 | 2.83 | 3.4 | 119.783 | 139.061 | 134.532 | H |
| I29.9 | 2 | 42.3 | 3_3381818_SNP | 38647437 | 2.54 | 3 | 129.53 | 141.934 | 124.785 | H |
| I30.1 | 4 | 71.8 | 4_56771900 | 3026374 | 5.01* | 9.5 | 139.317 | 141.143 | 104.326 | H |
| I30.2 | 7 | 83 | 4_8201938 | 3419851 | 4.99* | 9.5 | 102.79 | 115.559 | 140.853 | B |
| I30.3 | 2 | 95.5 | 1_8664417 | 1131098 | 3.15 | 5.8 | 132.024 | 138.732 | 111.619 | H |
| I30.4 | 10 | 30.7 | 2_8196429 | 42537207 | 3.11 | 5.8 | 117.215 | 141.25 | 126.429 | H |
| I30.5 | 8 | 43.5 | 2_56770831 | 54012080 | 2.5 | 4.6 | 115.851 | 138.722 | 127.793 | H |
| I31 | 4 | 76.4 | 1_8212370 | 2304527 | 6.4* | 16.6 | 163.136 | 168.998 | 120.392 | H |
| I32 | 4 | 71.8 | 4_56771900 | 3026374 | 7.95* | 20.2 | 160.719 | 168.122 | 111.858 | H |
| I33.1 | 4 | 71.8 | 4_56771900 | 3026374 | 9.67* | 22 | 125.11 | 146.647 | 78.2912 | H |
| I33.2 | 1 | 34.5 | 1_56771237 | 41207455 | 3.07 | 6.3 | 124.63 | 130.166 | 78.7711 | H |
| I33.3 | 9 | 22.2 | 1_3367403 | 34764819 | 2.88 | 5.9 | 92.8199 | 117.992 | 110.497 | H |
| I34.1 | 4 | 71.8 | 4_56771900 | 3026374 | 6.65* | 16.1 | 169.667 | 182.467 | 120.272 | H |
| I34.2 | 9 | 22.2 | 1_3367403 | 34764819 | 2.87 | 6.5 | 131.581 | 156.37 | 158.172 | B |
| I34.3 | 4 | 36.7 | 2_3375247 | 40411191 | 2.66 | 6 | 135.085 | 122.052 | 154.855 | B |
| I35.1 | 4 | 71.8 | 4_56771900 | 3026374 | 5.25* | 13.6 | 145.593 | 158.679 | 102.371 | H |
| I35.2 | 2 | 95.5 | 1_8664417 | 1131098 | 2.85 | 7.1 | 138.852 | 141.721 | 109.112 | H |
| I36.1 | 4 | 71.8 | 4_56771900 | 3026374 | 7.07* | 17.4 | 156.333 | 165.572 | 100.535 | H |
| I36.2 | 2 | 97.3 | 1_8181646 | 829245 | 3.64* | 8.5 | 138.953 | 152.745 | 117.915 | H |
| I36.3 | 4 | 44.8 | 1_8670627 | 8976921 | 3.31 | 7.7 | 106.569 | 111.9 | 150.299 | B |
| I37.1 | 4 | 71.8 | 4_56771900 | 3026374 | 4.98* | 12.8 | 141.534 | 152.316 | 91.6724 | H |
| I37.2 | 2 | 103.4 | 1_22459442 | 520142 | 4.36* | 11.1 | 131.749 | 146.909 | 101.458 | H |
| I38.1 | 2 | 103.4 | 1_22459442 | 520142 | 4.35* | 12.2 | 133.328 | 153.984 | 111.118 | H |
| I38.2 | 4 | 71.8 | 4_56771900 | 3026374 | 2.65 | 7.2 | 143.455 | 143.071 | 100.991 | A |
| I39.1 | 4 | 71.8 | 4_56771900 | 3026374 | 3.28 | 8.7 | 125.685 | 124.451 | 77.2188 | A |
| I39.2 | 1 | 25.3 | 1_8664503 | 19789605 | 2.78 | 7.3 | 123.8 | 122.953 | 79.1043 | A |
| I39.3 | 8 | 5.7 | 1_3370746 | 45849402 | 2.5 | 6.5 | 109.476 | 123.229 | 93.4276 | H |
| I40 | 4 | 71.8 | 4_56771900 | 3026374 | 3.15 | 10 | 151.549 | 157.909 | 97.3668 | H |
| I42 | 4 | 52.9 | 2_3366022 | 5485430 | 2.59 | 8.9 | 138.075 | 163.992 | 119.185 | H |
| I43 | 4 | 52.9 | 2_3366022 | 5485430 | 3.2 | 11.1 | 138.885 | 166.406 | 104.459 | H |
| I46 | 4 | 52.9 | 2_3366022 | 5485430 | 2.75 | 10.4 | 136.785 | 157.328 | 76.9739 | H |
| I49.1 | 1 | 9.4 | 1_3374599 | Chr02:42127879 | 3.49 | 12.9 | 131.65 | 142.263 | 86.2422 | H |
| I49.2 | 6 | 30.2 | 4_56770939 | 25282270 | 3.04 | 11.1 | 122.355 | 144.899 | 95.5372 | H |
| I51.1 | 4 | 39.2 | 2_56771074 | 15682230 | 3.77* | 15.2 | 184.495 | 150.044 | 220.885 | B |
| I51.2 | 11 | 10.6 | 1_56771366 | 50475544 | 2.52 | 9.8 | 216.62 | 173.892 | 188.759 | A |
| I54 | 11 | 46.5 | 1_3371773 | 4640458 | 3.72* | 16.8 | 153.991 | 114.52 | 189.269 | B |
| I55.1 | 11 | 46.5 | 1_3371773 | 4640458 | 3.74* | 15.6 | 134.14 | 100.643 | 174.407 | B |
| I55.2 | 6 | 24.1 | 2_3365849 | 23286803 | 2.67 | 10.8 | 166.966 | 189.045 | 141.547 | H |
| I59.1 | 11 | 42.6 | 4_3381244 | 5756845 | 3.64 | 17.2 | 154.384 | 103.266 | 145.683 | A |
| I59.2 | 3 | 57.4 | 2_3370492 | 40762545 | 2.79 | 12.8 | 164.55 | 186.922 | 135.643 | H |
| I60 | 11 | 42.6 | 4_3381244 | 5756845 | 2.46 | 14 | 170.44 | 124.846 | 192.516 | B |
| I61 | 1 | 19.9 | 2_8209039 | 7750012 | 2.63 | 15.7 | 173.529 | 148.736 | 84.914 | A |
| I64 | 3 | 83.3 | 1_3370597 | 50217899 | 3.25 | 22.1 | 160.552 | 117.957 | 197.037 | B |
| I65 | 3 | 78.6 | 2_8664362 | 48943476 | 2.77 | 19.2 | 158.176 | 114.756 | 198.75 | B |
| I66.1 | 2 | 42.3 | 3_3381818_SNP | 38647437 | 4.92* | 24.2 | 121.729 | 190.184 | 156.843 | H |
| I66.2 | 6 | 59.1 | 1_8207921 | 31192341 | 3.61 | 16.7 | 153.863 | 188.213 | 124.709 | H |
| I66.3 | 7 | 0 | 1_56771264 | 39920349 | 3.24 | 14.8 | 161.999 | 75.1139 | 116.572 | A |
| I71 | 3 | 30.8 | 4_56771453 | 3352480 | 3.77* | 37.4 | 143.027 | 168.238 | 268.151 | B |
| I75.1 | 2 | 12.7 | 2_8179068 | 48841227 | 4.99* | 46.7 | 149.66 | 204.391 | 118.805 | H |
| I75.2 | 6 | 41.6 | 1_22343793 | 27336359 | 2.88 | 22.3 | 135.8 | 82.8198 | 132.665 | A |
| I76 | 8 | 5.1 | 1_56771805 | 61133287 | 3.3 | 44.3 | 69.0919 | 165.187 | 141.397 | H |
| I78 | 9 | 16.7 | 1_56771415 | 35883048 | 3.31 | 51.6 | 72.5829 | 167.415 | 125.198 | H |
| I80.1 | 4 | 71.8 | 4_56771900 | 3026374 | 3.57 | 33 | 22.2974 | 59.5935 | 209.316 | B |
| I80.2 | 10 | 69.1 | 4_56771169 | 3398468 | 2.93 | 24.6 | 119.225 | 193.951 | 112.389 | H |

**Table S9.** Details of identified QTL within the four main QTL regions on Chr01, Chr02, Chr04 and Chr07. Table details the map position, peak QTL marker, genome position in the common bean reference genome (specified where markers blasted to a different chromosome than mapped to), LOD score, percentage of variation explained by the QTL (PVE), * signifies where a LOD score was significant (above genome wide significance), and estimated average values for the genotypes G12873 (mu_A), Midas (mu_B) and the HETs (mu_H) of identified QTL. These values are coloured to better assess allelic direction, with larger elongation values coloured darker green. Allelic direction for longer internodes is specified in the second last column.

| **QTL** | **Chromosome** | **CentiMorgan** | **Associated Marker** | **Genome Position (bp)** | **LOD** | **PVE (%)** | **mu_A** | **mu_H** | **mu_B** | **Allelic direction** | **Cluster** |
| --- | --- | --- | --- | --- | --- | --- | --- | --- | --- | --- | --- |
| I4.6 | 1 | 42.9 | 1_3370781 | 45286620 | 2.93 | 3.5 | 100.56 | 106.275 | 132.6 | B | **Chr01** |
| I5.2 | 1 | 42.9 | 1_3370781 | 45286620 | 5.43* | 6.4 | 197.948 | 201.231 | 235.457 | B |  |
| I6.4 | 1 | 34.5 | 1_56771237 | 41207455 | 2.51 | 3.6 | 233.98 | 219.4 | 192.237 | A |  |
| I7.1 | 1 | 34.5 | 1_56771237 | 41207455 | 7.37* | 14.1 | 230.907 | 222.642 | 160.943 | A |  |
| I8.3 | 1 | 42.9 | 1_3370781 | 45286620 | 5.96* | 9 | 183.16 | 169.889 | 101.457 | A |  |
| I9.1 | 1 | 42.9 | 1_3370781 | 45286620 | 7.04* | 10.1 | 172.12 | 159.679 | 87.493 | A |  |
| I14.2 | 1 | 47.3 | 1_3378797 | 46538386 | 3.09 | 5.9 | 129.506 | 132.23 | 84.9816 | H |  |
| I15.1 | 1 | 47.3 | 1_3378797 | 46538386 | 4.57* | 8.4 | 146.107 | 149.872 | 94.8513 | H |  |
| I16 | 1 | 47.3 | 1_3378797 | 46538386 | 5.01* | 11.7 | 145.562 | 143.608 | 78.9387 | A |  |
| I17 | 1 | 47.3 | 1_3378797 | 46538386 | 2.51 | 6.1 | 143.982 | 137.601 | 83.0019 | A |  |
| I18 | 1 | 47.3 | 1_3378797 | 46538386 | 3.79* | 9.2 | 149.186 | 139.808 | 69.2402 | A |  |
| I19.1 | 1 | 53.2 | 1_3380906 | 47899383 | 7.93* | 16.5 | 163.115 | 153.877 | 89.1504 | A |  |
| I20.3 | 1 | 47.3 | 1_3378797 | 46538386 | 2.8 | 5.8 | 236.728 | 217.979 | 106.706 | A |  |
| I21.1 | 1 | 47.3 | 1_3378797 | 46538386 | 5.35* | 10.4 | 173.528 | 170.78 | 48.8917 | A |  |
| I3.2 | 2 | 102.1 | 1_22342965 | 547948 | 5.28* | 7.2 | 39.2385 | 56.433 | 30.7805 | H | **Chr02** |
| I4.2 | 2 | 103.4 | 1_22459442 | 520142 | 5.47* | 6.7 | 123.752 | 151.437 | 109.408 | H |  |
| I5.5 | 2 | 102.1 | 1_22342965 | 547948 | 2.73 | 3.1 | 222.362 | 237.714 | 211.043 | H |  |
| I8.2 | 2 | 95.5 | 1_8664417 | 1131098 | 6.2* | 9.4 | 160.473 | 160.752 | 124.143 | H |  |
| I9.5 | 2 | 97.3 | 1_8181646 | 829245 | 4.16* | 5.8 | 142.142 | 140.697 | 117.471 | A |  |
| I24 | 2 | 103.4 | 1_22459442 | 520142 | 2.89 | 7.4 | 178.12 | 181.626 | 152.348 | H |  |
| I25.1 | 2 | 103.4 | 1_22459442 | 520142 | 5.35* | 10.9 | 146.334 | 154.428 | 114.951 | H |  |
| I26.1 | 2 | 97.3 | 1_8181646 | 829245 | 5.92* | 11.6 | 173.483 | 176.275 | 137.556 | H |  |
| I27.1 | 2 | 97.3 | 1_8181646 | 829245 | 5.26* | 10.3 | 149.61 | 152.903 | 119.522 | H |  |
| I29.2 | 2 | 97.3 | 1_8181646 | 829245 | 7.84* | 10 | 141.227 | 145.791 | 113.088 | H |  |
| I30.3 | 2 | 95.5 | 1_8664417 | 1131098 | 3.15 | 5.8 | 132.024 | 138.732 | 111.619 | H |  |
| I35.2 | 2 | 95.5 | 1_8664417 | 1131098 | 2.85 | 7.1 | 138.852 | 141.721 | 109.112 | H |  |
| I36.2 | 2 | 97.3 | 1_8181646 | 829245 | 3.64* | 8.5 | 138.953 | 152.745 | 117.915 | H |  |
| I37.2 | 2 | 103.4 | 1_22459442 | 520142 | 4.36* | 11.1 | 131.749 | 146.909 | 101.458 | H |  |
| I38.1 | 2 | 103.4 | 1_22459442 | 520142 | 4.35* | 12.2 | 133.328 | 153.984 | 111.118 | H |  |
| I8.5 | 4 | 71.8 | 4_56771900 | 3026374 | 3.28 | 4.8 | 155.776 | 156.356 | 128.84 | H | **Chr04** |
| I9.2 | 4 | 71.8 | 4_56771900 | 3026374 | 7.04* | 10.1 | 146.884 | 144.529 | 112.728 | A |  |
| I10.2 | 4 | 71.8 | 4_56771900 | 3026374 | 3.98* | 8 | 182.839 | 178.83 | 151.992 | A |  |
| I11.1 | 4 | 71.8 | 4_56771900 | 3026374 | 5.19* | 11.1 | 166.928 | 161.84 | 131.758 | A |  |
| I12 | 4 | 71.8 | 4_56771900 | 3026374 | 6.53* | 14.7 | 157.689 | 165.02 | 125.598 | H |  |
| I13.1 | 4 | 71.8 | 4_56771900 | 3026374 | 4.8* | 10.6 | 151.226 | 161.24 | 129.152 | H |  |
| I14.1 | 4 | 71.8 | 4_56771900 | 3026374 | 4.8* | 9.4 | 120.905 | 126.516 | 93.582 | H |  |
| I15.2 | 4 | 71.8 | 4_56771900 | 3026374 | 4.09* | 7.4 | 133.205 | 135.553 | 107.753 | H |  |
| I25.2 | 4 | 71.8 | 4_56771900 | 3026374 | 3.85* | 7.7 | 145.635 | 151.425 | 115.65 | H |  |
| I26.2 | 4 | 76.4 | 1_8212370 | 2304527 | 5.71* | 11.2 | 173.681 | 181.167 | 137.358 | H |  |
| I27.2 | 4 | 71.8 | 4_56771900 | 3026374 | 4.9* | 9.5 | 151.401 | 152.805 | 117.731 | H |  |
| I29.1 | 4 | 71.8 | 4_56771900 | 3026374 | 11.23* | 15 | 149.399 | 149.1 | 104.916 | A |  |
| I30.1 | 4 | 71.8 | 4_56771900 | 3026374 | 5.01* | 9.5 | 139.317 | 141.143 | 104.326 | H |  |
| I31 | 4 | 76.4 | 1_8212370 | 2304527 | 6.4* | 16.6 | 163.136 | 168.998 | 120.392 | H |  |
| I32 | 4 | 71.8 | 4_56771900 | 3026374 | 7.95* | 20.2 | 160.719 | 168.122 | 111.858 | H |  |
| I33.1 | 4 | 71.8 | 4_56771900 | 3026374 | 9.67* | 22 | 125.11 | 146.647 | 78.2912 | H |  |
| I34.1 | 4 | 71.8 | 4_56771900 | 3026374 | 6.65* | 16.1 | 169.667 | 182.467 | 120.272 | H |  |
| I35.1 | 4 | 71.8 | 4_56771900 | 3026374 | 5.25* | 13.6 | 145.593 | 158.679 | 102.371 | H |  |
| I36.1 | 4 | 71.8 | 4_56771900 | 3026374 | 7.07* | 17.4 | 156.333 | 165.572 | 100.535 | H |  |
| I37.1 | 4 | 71.8 | 4_56771900 | 3026374 | 4.98* | 12.8 | 141.534 | 152.316 | 91.6724 | H |  |
| I38.2 | 4 | 71.8 | 4_56771900 | 3026374 | 2.65 | 7.2 | 143.455 | 143.071 | 100.991 | A |  |
| I39.1 | 4 | 71.8 | 4_56771900 | 3026374 | 3.28 | 8.7 | 125.685 | 124.451 | 77.2188 | A |  |
| I40 | 4 | 71.8 | 4_56771900 | 3026374 | 3.15 | 10 | 151.549 | 157.909 | 97.3668 | H |  |
| I1.1 | 7 | 72 | 1_56771959 | 6145270 | 3.98* | 5.8 | 15.1604 | 16.0239 | 17.9867 | B | **Chr07** |
| I2.2 | 7 | 60.3 | 1_56770837 | scaffold_16 | 3.14 | 4.8 | 14.9238 | 18.9116 | 23.7035 | B |  |
| I3.1 | 7 | 60.3 | 1_56770837 | scaffold_16 | 5.59* | 7.6 | 19.4133 | 32.5408 | 50.6057 | B |  |
| I4.1 | 7 | 67 | 1_56771207 | 7430931 | 8.36* | 10.5 | 83.5435 | 128.945 | 149.616 | B |  |
| I5.1 | 7 | 67 | 1_56771207 | 7430931 | 9.38* | 11.4 | 179.779 | 222.146 | 253.627 | B |  |

**Table S10.** Details of identified candidates within the four main QTL regions on Chr01, Chr02, Chr04 and Chr07 based on identified links to hypocotyl or stem elongation in the literature. Accompanies **Fig. 3** in the main text. Candidate genes in bold are considered the most relevant, and flowering candidates are denoted by blue text. References for the relevant literature is provided below the table.

| **Cluster** | **2-LOD region** | **Candidate gene** | **Annotated function of candidate gene** | **Potential link to internode elongation** |
| --- | --- | --- | --- | --- |
| ***Pv01*** | Phvul.001G177400 - Phvul.001G224200 (472 genes) | Phvul.001G174200  (Just outside 2-LOD region) | *Protein REVEILLE 4-RELATED* | Represses hypocotyl length in *Arabidopsis* (Gray et al., 2017) |
|  |  | Phvul.001G174400  (Just outside 2-LOD region) | *AP2-like factor (AP2)* | *APETALA2* promotes stem elongation in Barley (Patil et al., 2019) |
|  |  | Phvul.001G182200 to Phvul.001G182900 | *UDP-glucosyl transferase 73C brassinosteroid* | Brassinosteriods promote stem elongation in closely related soybean (Chen et al., 2021) |
|  |  | Phvul.001G185500 | *Cytochrome P450 CYP2 subfamily* | Genes in Cytochrome P450 can be part of the GA metabolic pathway and are known to influence stem elongation, such as in rice (Itoh et al., 2004) & pea (Davidson et al., 2004) |
|  |  | Phvul.001G187500 | *Growth-Regulating Factor 3-Related* | Ectopic expression of *OsGRF1* in Arabidopsis reduced stem elongation (van der Knaap et al., 2000) |
|  |  | Phvul.001G188500. | *Cytochrome P450 CYP4/CYP19/CYP26 subfamilies* | Genes in Cytochrome P450 can be part of the GA metabolic pathway and are known to influence stem elongation, such as in rice (Itoh et al., 2004) & pea (Davidson et al., 2004) |
|  |  | **Phvul.001G189200** | ***TFLY1 (FIN)*** | **Growth habit gene, conferring determinacy in common bean (Repinski et al., 2012)** |
|  |  | Phvul.001G189400 | *Abscisic Acid-Insensitive 5* | ABA is known to inhibit stem elongation (Lorrai et al., 2018) |
|  |  | Phvul.001G195800 | *BEL1-LIKE Homeodomain Protein 1-Related* | The *BEL1-like* homeodomain protein *OsBLH4* promotes stem elongation in rice (Cao et al., 2024) |
|  |  | Phvul.001G196800 | *Nuclear Transcription Factor Y Subunit A-10-Related* | *NF-Y* has been demonstrated to regulate hypocotyl elongation in Arabidopsis (Zhao et al., 2016) |
|  |  | Phvul.001G207000 | *PHYTOCHROME A-ASSOCIATED F-BOX PROTEIN (EID1-like)* | *EID1* involved in hypocotyl elongation in *Arabidopsis* (Dieterle et al., 2001) |
|  |  | Phvul.001G213100 | *CYTOCHROME C OXIDASE SUBUNIT 5C-2-RELATED* | *Cytochrome c* (*CYTC*) deficient plants exhibit lower levels of active GA, and *cytc* mutant plants exhibit reduced hypocotyl elongation (Racca et al., 2018) |
|  |  | Phvul.001G213600 | *WRKY DNA-binding domain* | WRKY proteins known to influence stem elongation, such as in rice (Zhang et al., 2011) |
|  |  | Phvul.001G214400 | *WRKY TRANSCRIPTION FACTOR 23-RELATED* |  |
|  |  | Phvul.001G218500 | *WRKY DNA-binding domain* |  |
|  |  | Phvul.001G218700 | *Auxin-Responsive Protein IAA20-Related* | Auxin is known to regulate stem elongation, with several IAA proteins inhibiting elongation in Arabidopsis (Reed et al., 2018) |
|  |  | Phvul.001G218800 | *Phytochrome-interacting factor 3 (PIF3)* | Promotes hypocotyl elongation in Arabidopsis (Kim et al., 2003) |
|  |  | **Phvul.001G221100** | ***PHYA3 (Ppd)*** | **Flowering time gene, demonstrated to regulate stem elongation in Medicago (Jaudal et al., 2020)** |
|  |  | Phvul.001G230500  (Just outside 2-LOD region) | *DELLA protein* | Well known negative regulator of stem elongation in plants, such as in rice (Ikeda et al., 2001) |
| ***Pv02*** | Phvul.002G004500 - Phvul.002G019800 (154 genes) | Phvul.002G007600 | *Leucine Rich Repeat ;LRRNT_2* | Leucine-rich repeat receptor-like kinases are numerous, some of which like *ERECTA (ER)* and *ER-LIKE* have been shown to regulate stem elongation in Arabidopsis (Qu et al., 2017) |
|  |  | Phvul.002G007800 | *LRR RECEPTOR-LIKE; HSL2* |  |
|  |  | Phvul.002G009100 | *AP2-like factor (AP2)* | *APETALA2* promotes stem elongation in Barley (Patil et al., 2019) |
|  |  | Phvul.002G010700 | *Protein SHORT-ROOT* | Promotes hypocotyl elongation in *Arabidopsis* (Dhar et al., 2022) |
|  |  | Phvul.002G013800 | *ESTERASE KARRIKIN INSENSITIVE 2 (KAI2)-Related* | Regulates hypocotyl elongation in *Arabidopsis* (Guercio et al., 2024) |
|  |  | Phvul.002G014400 | *Auxin Responsive GH3 gene family* | *GH3* gene family has been implicated in hypocotyl elongation in *Arabidopsis* (Takase et al., 2004) |
|  |  | Phvul.002G016100 | *WRKY Transcription Factor 15-Related* | WRKY proteins known to influence stem elongation, such as in rice (Zhang et al., 2011) |
|  |  | **Phvul.002G016900** | ***APETALA 2 (AP2)*** | ***APETALA2* promotes stem elongation in Barley (Patil et al., 2019)** |
| ***Pv04*** | Phvul.004G014200 - Phvul.004G028500 (160 genes) | Phvul.004G015000 | *Leucine Rich Repeat (LRR_2) // F-box domain (F-box) FBD (FBD)* | Leucine-rich repeat receptor-like kinases are numerous, some of which like *ERECTA (ER)* and *ER-LIKE* have been shown to regulate stem elongation in Arabidopsis (Qu et al., 2017) |
|  |  | Phvul.004G015100 | *Leucine Rich Repeat (LRR_2) // F-box domain (F-box) FBD (FBD)* |  |
|  |  | Phvul.004G015600 | *LEUCINE-RICH REPEAT-CONTAINING PROTEIN* |  |
|  |  | Phvul.004G015732 | *LEUCINE-RICH REPEAT-CONTAINING PROTEIN* |  |
|  |  | Phvul.004G015800 | *LEUCINE-RICH REPEAT-CONTAINING PROTEIN* |  |
|  |  | Phvul.004G015900 | *LEUCINE-RICH REPEAT-CONTAINING PROTEIN* |  |
|  |  | Phvul.004G016000 | *LEUCINE-RICH REPEAT-CONTAINING PROTEIN* |  |
|  |  | Phvul.004G016300 | *Leucine Rich Repeat (LRR_2) // F-box domain (F-box) FBD (FBD)* |  |
|  |  | Phvul.004G016400 | *Leucine Rich Repeat (LRR_2) // F-box domain (F-box) FBD (FBD)* |  |
|  |  | Phvul.004G016532 | *LEUCINE-RICH REPEAT-CONTAINING PROTEIN* |  |
|  |  | Phvul.004G016600 | *Leucine Rich Repeat (LRR_2) // F-box domain (F-box) FBD (FBD)* |  |
|  |  | Phvul.004G017100 | *BIDIRECTIONAL SUGAR TRANSPORTER SWEET4-RELATED* | Sugars Will Eventually Be Exported Transporters (SWEETs) play many roles in plant growth, while no direct link, a role in promoting stem elongation is feasible. |
|  |  | Phvul.004G017200 | *BIDIRECTIONAL SUGAR TRANSPORTER SWEET4-RELATED* |  |
|  |  | Phvul.004G017300 | *BIDIRECTIONAL SUGAR TRANSPORTER SWEET4-RELATED* |  |
|  |  | Phvul.004G017400 | *BIDIRECTIONAL SUGAR TRANSPORTER SWEET4-RELATED* |  |
|  |  | Phvul.004G017500 | *HOMEOBOX-LEUCINE ZIPPER PROTEIN ATHB-54-RELATED* | ATHB genes have been implicated in internode elongation in Arabidopsis (Baima et al., 2001) |
|  |  | Phvul.004G021100 | *Cytochrome P450 CYP2 subfamily* | Genes in Cytochrome P450 can be part of the GA metabolic pathway and are known to influence stem elongation, such as in rice (Itoh et al., 2004) & pea (Davidson et al., 2004) |
|  |  | Phvul.004G021200 | *Cytochrome P450 CYP2 subfamily* |  |
|  |  | Phvul.004G021300 | *Cytochrome P450 CYP2 subfamily* |  |
|  |  | Phvul.004G021400 | *Cytochrome P450 CYP2 subfamily* |  |
|  |  | Phvul.004G021500 | *Cytochrome P450 CYP2 subfamily* |  |
|  |  | Phvul.004G021600 | *Cytochrome P450 CYP2 subfamily* |  |
|  |  | Phvul.004G021700 | *Cytochrome P450 CYP2 subfamily* |  |
|  |  | Phvul.004G021800 | *Cytochrome P450 CYP2 subfamily* |  |
|  |  | Phvul.004G021900 | *Cytochrome P450 CYP2 subfamily* |  |
|  |  | Phvul.004G022000 | *Cytochrome P450 CYP2 subfamily* |  |
|  |  | Phvul.004G023066 | *ABC TRANSPORTER B FAMILY MEMBER 15-RELATED* | ABC transporter gene AtPGP1 promotes hypocotyl elongation (Sidler et al., 1998) |
|  |  | Phvul.004G023600 | *Leucine rich repeat N-terminal domain (LRRNT_2) // Leucine rich repeat (LRR_8)* | Leucine-rich repeat receptor-like kinases are numerous, some of which like *ERECTA (ER)* and *ER-LIKE* have been shown to regulate stem elongation in Arabidopsis (Qu et al., 2017) |
|  |  | Phvul.004G023700 | *Leucine Rich Repeat (LRR_1) // Protein tyrosine kinase (Pkinase_Tyr) // Leucine rich repeat N-terminal domain (LRRNT_2)* |  |
|  |  | Phvul.004G023800 | *CLAVATA3/ESR (CLE)-RELATED PROTEIN 25* | Overexpression of CLE genes resulted in dwarf plants (Strabala et al., 2006) |
|  |  | Phvul.004G024200 | *MYB-LIKE DNA-BINDING PROTEIN MYB* | MYB-Like genes are numerous, many MYB genes have been implicated to regulate plant height (e.g. (Wu et al., 2018)) |
|  |  | Phvul.004G026500 | *AUXIN EFFLUX CARRIER COMPONENT 5-RELATED* | Auxin transport is critical to hypocotyl elongation (Jensen et al., 1998) |
|  |  | Phvul.004G026700 | *F-BOX AND WD40 DOMAIN PROTEIN* | WD40 repeat domain is found in many proteins, including *CONSTITUTIVE PHOTOMORPHOGENIC 1 (COP1)* in which this domain was found to be critical to hypocotyl regulation (Malakar et al., 2025) |
|  |  | Phvul.004G027500 | *HOMEOBOX-LEUCINE ZIPPER PROTEIN ATHB-20-RELATED* | ATHB genes have been implicated in internode elongation in Arabidopsis (Baima et al., 2001) |
|  |  | Phvul.004G027700 | *BHLH TRANSCRIPTION FACTOR PRE1-RELATED* | PRE1 promotes stem elongation in Arabidopsis and rice (Zhang et al., 2009) |
|  |  | Phvul.004G027800 | *ABC TRANSPORTER B FAMILY MEMBER 19* | ABC transporter gene AtPGP1 promotes hypocotyl elongation (Sidler et al., 1998) |
|  |  | Phvul.004G027900 | *Cullin 3 (CUL3)* | Culin genes have been implicated in root elongation (Moon et al., 2007; Thomann et al., 2009) |
|  |  | Phvul.004G028500 | *MYB-LIKE DNA-BINDING PROTEIN MYB* | MYB-Like genes are numerous, many MYB genes have been implicated to regulate plant height (e.g. (Wu et al., 2018)) |
|  |  | Phvul.004G046601  (Just outside 2-LOD region) | *ZINC FINGER PROTEIN CONSTANS-RELATED (COL2)* | Flowering time gene in common bean. |
| ***Pv07*** | Phvul.007G041400 - Phvul.007G173700 (1186 genes) | Phvul.007G052000 | *Gibberellin 2-Beta-Dioxygenase7-RELATED (GA2ox7)* | Gibberellin is a long-known promoter of stem elongation, including the legumes pea, soybean and Medicago (Beagley & Weller, 2024) |
|  |  | Phvul.007G061000 | *RING ZINC FINGER PROTEIN* | RING-domain zinc finger proteins have been previously implicated in hypocotyl elongation (Chen & Ni, 2006) |
|  |  | **Phvul.007G061400** | ***Divergent CCT motif; PEAPOD ortholog*** | **Regulates hypocotyl length in Arabidopsis (White, 2022)** |
|  |  | Phvul.007G062000 | *EARLY LIGHT-INDUCED PROTEIN 1* | No direct link to stem elongation, however early light-induced protein (*ELIP*) genes have been shown to be regulated by *LONG HYPOCOTYL5 (HY5)* (Hayami et al., 2015) |
|  |  | Phvul.007G063200 | *LRR RECEPTOR-LIKE SERINE/THREONINE-PROTEIN KINASE ERL1-RELATED* | Leucine-rich repeat receptor-like kinases are numerous, some of which like *ERECTA (ER)* and *ER-LIKE* have been shown to regulate stem elongation in Arabidopsis (Qu et al., 2017) |
|  |  | Phvul.007G064800 | *Gibberellin 20-Oxidase 2 (GA20ox2) // Gibberellin-44 dioxygenase* | Gibberellin is a long-known promoter of stem elongation, including the legumes pea, soybean and Medicago (Beagley & Weller, 2024) |
|  |  | Phvul.007G065100 | *MADS Box Protein* | MADS Box transcription factors influence various aspects of development, including stem elongation, such as *SOC1* in Medicago (Jaudal et al., 2018) |
|  |  | Phvul.007G065550 | *MADS-Box Protein SOC1* | *SOC1* demonstrated to influence stem elongation in Medicago (Jaudal et al., 2018) |
|  |  | Phvul.007G067700 | *Leucine-rich repeat (LLR), protein tyrosine kinase* | Leucine-rich repeat receptor-like kinases are numerous, some of which like *ERECTA (ER)* and *ER-LIKE* have been shown to regulate stem elongation in Arabidopsis (Qu et al., 2017) |
|  |  | Phvul.007G071400 | *CYTOCHROME P450* | Genes in Cytochrome P450 can be part of the GA metabolic pathway and are known to influence stem elongation, such as in rice (Itoh et al., 2004) & pea (Davidson et al., 2004) |
|  |  | Phvul.007G071500 | *CYTOCHROME P450* |  |
|  |  | Phvul.007G074500 | *PROTEIN REVEILLE 3-RELATED* | Represses hypocotyl length in *Arabidopsis* (Gray et al., 2017) |
|  |  | Phvul.007G083500 | *GIGANTEA (GI)* | Regulates hypocotyl length in *Arabidopsis* (Mishra & Panigrahi, 2015) |
|  |  | Phvul.007G086300 | *LEUCINE-RICH REPEAT-CONTAINING PROTEIN // SUBFAMILY NOT NAMED* | Leucine-rich repeat receptor-like kinases are numerous, some of which like *ERECTA (ER)* and *ER-LIKE* have been shown to regulate stem elongation in Arabidopsis (Qu et al., 2017) |
|  |  | Phvul.007G104800 | *CYTOCHROME P450 77A4-RELATED* | Genes in Cytochrome P450 can be part of the GA metabolic pathway and are known to influence stem elongation, such as in rice (Itoh et al., 2004) & pea (Davidson et al., 2004) |
|  |  | Phvul.007G108200 | *PROTEIN LIGHT-DEPENDENT SHORT HYPOCOTYLS 1-RELATED* | *Light dependent short hypocotyl (LSH)* genes have been shown to repress hypocotyl elongation in Arabidopsis (Lee et al., 2020) |
|  |  | Phvul.007G108900 | *cryptochrome 2 (CRY2)* | Implicated in hypocotyl elongation in *Arabidopsis* (Jia et al., 2014) |
|  |  | Phvul.007G147100 | *BLUE-LIGHT PHOTORECEPTOR PHR2* | *PHR (Photolyase Homologous Region)* domains are in *Cryptochromes (CRYs)*. *PHR* overexpressing lines exhibited long-hypocotyls in *Arabidopsis* (Liu et al., 2020) |
|  |  | Phvul.007G156200 | *TRANSCRIPTION FACTOR PIF4-RELATED* | *PHYTOCHROME INTERACTING FACTORs (PIFs)* are well established to promote hypocotyl elongation (Choi & Oh, 2016) |
|  |  | Phvul.007G162800 | *PROTEIN REVEILLE 4-RELATED* | Represses hypocotyl length in *Arabidopsis* (Gray et al., 2017) |
|  |  | Phvul.007G163100 | *NUCLEAR TRANSCRIPTION FACTOR Y SUBUNIT B-2-RELATED* | *NF-Y* has been demonstrated to regulate hypocotyl elongation in Arabidopsis (Zhao et al., 2016) |

**Literature referenced in Supplementary Table S10:**

**Baima, S., Possenti, M., Matteucci, A., Wisman, E., Altamura, M. M., Ruberti, I., & Morelli, G.** (2001). The arabidopsis ATHB-8 HD-zip protein acts as a differentiation-promoting transcription factor of the vascular meristems. *Plant Physiol, 126*(2), 643-655. doi:<https://doi.org/10.1104/pp.126.2.643>

**Beagley, C. J., & Weller, J. L.** (2024). Genetic control of stem elongation in legume crops and its potential relevance. *Crop Science*. doi:<https://doi.org/10.1002/csc2.21283>

**Cao, C., Guo, S., Deng, P.*, et al.*** (2024). The BEL1-like homeodomain protein OsBLH4 regulates rice plant height, grain number, and heading date by repressing the expression of OsGA2ox1. *The plant journal, 119*(3), 1369-1385. doi:<https://doi.org/10.1111/tpj.16857>

**Chen, L. M., Yang, H. L., Fang, Y. S.*, et al.*** (2021). Overexpression of GmMYB14 improves high-density yield and drought tolerance of soybean through regulating plant architecture mediated by the brassinosteroid pathway. *Plant Biotechnology Journal, 19*(4), 702-716. doi:<https://doi.org/10.1111/pbi.13496>

**Chen, M., & Ni, M.** (2006). RFI2, a RING-domain zinc finger protein, negatively regulates CONSTANS expression and photoperiodic flowering. *The plant journal, 46*(5), 823-833. doi:<https://doi.org/10.1111/j.1365-313X.2006.02740.x>

**Choi, H., & Oh, E.** (2016). PIF4 Integrates Multiple Environmental and Hormonal Signals for Plant Growth Regulation in Arabidopsis. *Molecules and Cells, 39*(8), 587-593. doi:<https://doi.org/10.14348/molcells.2016.0126>

**Davidson, S. E., Smith, J. J., Helliwell, C. A., Poole, A. T., & Reid, J. B.** (2004). The pea gene LH encodes ent-kaurene oxidase. *Plant physiology, 134*(3), 1123-1134. doi:<https://doi.org/10.1104/pp.103.032706>

**Dhar, S., Kim, J., Yoon, E. K., Jang, S., Ko, K., & Lim, J.** (2022). SHORT-ROOT Controls Cell Elongation in the Etiolated Arabidopsis Hypocotyl. *Molecules and Cells, 45*(4), 243-256. doi:<https://doi.org/10.14348/molcells.2021.5008>

**Dieterle, M., Zhou, Y. C., Schäfer, E., Funk, M., & Kretsch, T.** (2001). EID1, an F-box protein involved in phytochrome A-specific light signaling. *Genes Dev, 15*(8), 939-944. doi:<https://doi.org/10.1101/gad.197201>

**Gray, J. A., Shalit-Kaneh, A., Chu, D. N., Hsu, P. Y., & Harmer, S. L.** (2017). The REVEILLE Clock Genes Inhibit Growth of Juvenile and Adult Plants by Control of Cell Size. *Plant physiology, 173*(4), 2308-2322. doi:<https://doi.org/10.1104/pp.17.00109>

**Guercio, A. M., Gilio, A. K., Pawlak, J., & Shabek, N.** (2024). Structural insights into rice KAI2 receptor provide functional implications for perception and signal transduction. *J Biol Chem, 300*(8), 107593. doi:<https://doi.org/10.1016/j.jbc.2024.107593>

**Hayami, N., Sakai, Y., Kimura, M.*, et al.*** (2015). The Responses of Arabidopsis Early Light-Induced Protein2 to Ultraviolet B, High Light, and Cold Stress Are Regulated by a Transcriptional Regulatory Unit Composed of Two Elements  *Plant physiology, 169*(1), 840-855. doi:<https://doi.org/10.1104/pp.15.00398>

**Ikeda, A., Ueguchi-Tanaka, M., Sonoda, Y., Kitano, H., Koshioka, M., Futsuhara, Y., Matsuoka, M., & Yamaguchi, J.** (2001). slender rice, a constitutive gibberellin response mutant, is caused by a null mutation of the SLR1 gene, an ortholog of the height-regulating gene GAI/RGA/RHT/D8. *The Plant cell, 13*(5), 999-1010. doi:<https://doi.org/10.2307/3871359>

**Itoh, H., Tatsumi, T., Sakamoto, T., Otomo, K., Toyomasu, T., Kitano, H., Ashikari, M., Ichihara, S., & Matsuoka, M.** (2004). A Rice Semi-Dwarf Gene, Tan-Ginbozu (D35), Encodes the Gibberellin Biosynthesis Enzyme, ent-Kaurene Oxidase. *Plant Molecular Biology, 54*(4), 533-547. doi:<https://doi.org/10.1023/B:PLAN.0000038261.21060.47>

**Jaudal, M., Wen, J. Q., Mysore, K. S., & Putterill, J.** (2020). Medicago PHYA promotes flowering, primary stem elongation and expression of flowering time genes in long days. *BMC Plant Biology, 20*(1). doi:<https://doi.org/10.1186/s12870-020-02540-y>

**Jaudal, M., Zhang, L. L., Che, C., Li, G. F., Tang, Y. H., Wen, J. Q., Mysore, K. S., & Putterill, J.** (2018). A SOC1-like gene MtSOC1a promotes flowering and primary stem elongation in Medicago. *Journal of Experimental Botany, 69*(20), 4867-4880. doi:<https://doi.org/10.1093/jxb/ery284>

**Jensen, P. J., Hangarter, R. P., & Estelle, M.** (1998). Auxin transport is required for hypocotyl elongation in light-grown but not dark-grown Arabidopsis. *Plant Physiol, 116*(2), 455-462. doi:<https://doi.org/10.1104/pp.116.2.455>

**Jia, K.-P., Luo, Q., He, S.-B., Lu, X.-D., & Yang, H.-Q.** (2014). Strigolactone-Regulated Hypocotyl Elongation Is Dependent on Cryptochrome and Phytochrome Signaling Pathways in <em>Arabidopsis</em>. *Molecular Plant, 7*(3), 528-540. doi:<https://doi.org/10.1093/mp/sst093>

**Kim, J., Yi, H., Choi, G., Shin, B., Song, P. S., & Choi, G.** (2003). Functional characterization of phytochrome interacting factor 3 in phytochrome-mediated light signal transduction. *The Plant cell, 15*(10), 2399-2407. doi:<https://doi.org/10.1105/tpc.014498>

**Lee, M., Dong, X., Song, H., Yang, J. Y., Kim, S., & Hur, Y.** (2020). Molecular characterization of Arabidopsis thaliana LSH1 and LSH2 genes. *Genes & Genomics, 42*(10), 1151-1162. doi:<https://doi.org/10.1007/s13258-020-00985-x>

**Liu, Q., Su, T., He, W.*, et al.*** (2020). Photooligomerization Determines Photosensitivity and Photoreactivity of Plant Cryptochromes. *Molecular Plant, 13*(3), 398-413. doi:<https://doi.org/10.1016/j.molp.2020.01.002>

**Lorrai, R., Boccaccini, A., Ruta, V., Possenti, M., Costantino, P., & Vittorioso, P.** (2018). Abscisic acid inhibits hypocotyl elongation acting on gibberellins, DELLA proteins and auxin. *AoB Plants, 10*(5). doi:<https://doi.org/10.1093/aobpla/ply061>

**Malakar, B. C., Escudero, C. M., Sethi, V., Upadhyaya, G., Gangappa, S. N., & Botto, J. F.** (2025). The COP1W467 tryptophan residue in the WD40 domain is essential for light- and temperature-mediated hypocotyl growth and flowering in Arabidopsis. *The plant journal, 121*(4), e70051. doi:<https://doi.org/10.1111/tpj.70051>

**Mishra, P., & Panigrahi, K. C.** (2015). GIGANTEA - an emerging story. *Front Plant Sci, 6*, 8. doi:<https://doi.org/10.3389/fpls.2015.00008>

**Moon, J., Zhao, Y., Dai, X., Zhang, W., Gray, W. M., Huq, E., & Estelle, M.** (2007). A new CULLIN 1 mutant has altered responses to hormones and light in Arabidopsis. *Plant Physiol, 143*(2), 684-696. doi:<https://doi.org/10.1104/pp.106.091439>

**Patil, V., McDermott, H. I., McAllister, T.*, et al.*** (2019). APETALA2 control of barley internode elongation. *Development, 146*(11). doi:<https://doi.org/10.1242/dev.170373>

**Qu, X., Zhao, Z., & Tian, Z.** (2017). ERECTA Regulates Cell Elongation by Activating Auxin Biosynthesis in Arabidopsis thaliana. *Frontiers in Plant Science, Volume 8 - 2017*. doi:10.3389/fpls.2017.01688

**Racca, S., Welchen, E., Gras, D. E., Tarkowská, D., Turečková, V., Maurino, V. G., & Gonzalez, D. H.** (2018). Interplay between cytochrome c and gibberellins during Arabidopsis vegetative development. *The plant journal, 94*(1), 105-121. doi:<https://doi.org/10.1111/tpj.13845>

**Reed, J. W., Wu, M.-F., Reeves, P. H., Hodgens, C., Yadav, V., Hayes, S., & Pierik, R.** (2018). Three Auxin Response Factors Promote Hypocotyl Elongation. *Plant physiology, 178*(2), 864-875. doi:<https://doi.org/10.1104/pp.18.00718>

**Repinski, S. L., Kwak, M., & Gepts, P.** (2012). The common bean growth habit gene PvTFL1y is a functional homolog of Arabidopsis TFL1. *Theoretical and Applied Genetics, 124*(8), 1539-1547. doi:<https://doi.org/10.1007/s00122-012-1808-8>

**Sidler, M., Hassa, P., Hasan, S., Ringli, C., & Dudler, R.** (1998). Involvement of an ABC Transporter in a Developmental Pathway Regulating Hypocotyl Cell Elongation in the Light. *The Plant cell, 10*(10), 1623-1636. doi:<https://doi.org/10.1105/tpc.10.10.1623>

**Strabala, T. J., O'Donnell P, J., Smit, A. M.*, et al.*** (2006). Gain-of-function phenotypes of many CLAVATA3/ESR genes, including four new family members, correlate with tandem variations in the conserved CLAVATA3/ESR domain. *Plant Physiol, 140*(4), 1331-1344. doi:<https://doi.org/10.1104/pp.105.075515>

**Takase, T., Nakazawa, M., Ishikawa, A., Kawashima, M., Ichikawa, T., Takahashi, N., Shimada, H., Manabe, K., & Matsui, M.** (2004). ydk1-D, an auxin-responsive GH3 mutant that is involved in hypocotyl and root elongation. *The plant journal, 37*(4), 471-483. doi:<https://doi.org/10.1046/j.1365-313X.2003.01973.x>

**Thomann, A., Lechner, E., Hansen, M., Dumbliauskas, E., Parmentier, Y., Kieber, J., Scheres, B., & Genschik, P.** (2009). Arabidopsis CULLIN3 genes regulate primary root growth and patterning by ethylene-dependent and -independent mechanisms. *PLoS Genet, 5*(1), e1000328. doi:<https://doi.org/10.1371/journal.pgen.1000328>

**van der Knaap, E., Kim, J. H., & Kende, H.** (2000). A novel gibberellin-induced gene from rice and its potential regulatory role in stem growth. *Plant physiology, 122*(3), 695-704. doi:<https://doi.org/10.1104/pp.122.3.695>

**Voorrips, R.** (2002). MapChart: software for the graphical presentation of linkage maps and QTLs. *Journal of heredity, 93*(1), 77-78. doi:<https://doi.org/10.1093/jhered/93.1.77>

**White, D. W. R.** (2022). PEAPOD repressors modulate and coordinate developmental responses to light intensity in Arabidopsis. *New Phytologist, 235*(4), 1470-1485. doi:<https://doi.org/10.1111/nph.18198>

**Wu, P., Peng, M., Li, Z.*, et al.*** (2018). DRMY1, a Myb-Like Protein, Regulates Cell Expansion and Seed Production in Arabidopsis thaliana. *Plant and Cell Physiology, 60*(2), 285-302. doi:<https://doi.org/10.1093/pcp/pcy207>

**Zhang, C.-Q., Xu, Y., Lu, Y., Yu, H.-X., Gu, M.-H., & Liu, Q.-Q.** (2011). The WRKY transcription factor OsWRKY78 regulates stem elongation and seed development in rice. *Planta, 234*(3), 541-554. doi:<https://doi.org/10.1007/s00425-011-1423-y>

**Zhang, L. Y., Bai, M. Y., Wu, J.*, et al.*** (2009). Antagonistic HLH/bHLH transcription factors mediate brassinosteroid regulation of cell elongation and plant development in rice and Arabidopsis. *The Plant cell, 21*(12), 3767-3780. doi:<https://doi.org/10.1105/tpc.109.070441>

**Zhao, H., Wu, D., Kong, F., Lin, K., Zhang, H., & Li, G.** (2016). The Arabidopsis thaliana Nuclear Factor Y Transcription Factors. *Front Plant Sci, 7*, 2045. doi:<https://doi.org/10.3389/fpls.2016.02045>
